# Prevalence of G6PD deficiency in selected populations from two previously high malaria endemic areas of Sri Lanka

**DOI:** 10.1371/journal.pone.0171208

**Published:** 2017-02-02

**Authors:** Sharmini Gunawardena, G. M. G. Kapilananda, Dilhani Samarakoon, Sashika Maddevithana, Sulochana Wijesundera, Lallindra V. Goonaratne, Nadira D. Karunaweera

**Affiliations:** 1 Department of Parasitology, Faculty of Medicine, University of Colombo, Colombo, Sri Lanka; 2 Department of Biochemistry, Faculty of Medicine, University of Colombo, Colombo, Sri Lanka; 3 Department of Pathology, Faculty of Medicine, University of Colombo, Colombo, Sri Lanka; Centro de Pesquisas Rene Rachou, BRAZIL

## Abstract

Glucose-6-Phosphate Dehydrogenase (G6PD) enzyme deficiency is known to offer protection against malaria and an increased selection of mutant genes in malaria endemic regions is expected. However, anti-malarial drugs such as primaquine can cause haemolytic anaemia in persons with G6PD deficiency. We studied the extent of G6PD deficiency in selected persons attending Teaching Hospitals of Anuradhapura and Kurunegala, two previously high malaria endemic districts in Sri Lanka. A total of 2059 filter-paper blood spots collected between November 2013 and June 2014 were analysed for phenotypic G6PD deficiency using the modified WST-8/1-methoxy PMS method. Each assay was conducted with a set of controls and the colour development assessed visually as well as with a microplate reader at OD^450-630^nm. Overall, 142/1018 (13.95%) and 83/1041 (7.97%) were G6PD deficient in Anuradhapura and Kurunegala districts respectively. The G6PD prevalence was significantly greater in Anuradhapura when compared to Kurunegala (P<0.0001). Surprisingly, females were equally affected as males in each district: 35/313 (11.18%) males and 107/705 (15.18%) females were affected in Anuradhapura (P = 0.089); 25/313 (7.99%) males and 58/728 (7.97%) females were affected in Kurunegala (P = 0.991). Prevalence was greater among females in Anuradhapura than in Kurunegala (P<0.05), while no such difference was observed between the males (P>0.05). Severe deficiency (<10% normal) was seen among 28/1018 (2.75%) in Anuradhapura (7 males; 21 females) and 17/1041 (1.63%) in Kurunegala (7 males; 10 females). Enzyme activity between 10–30% was observed among 114/1018 (11.20%; 28 males; 86 females) in Anuradhapura while it was 66/1041 (6.34%; 18 males; 48 females) in Kurunegala. Screening and educational programmes for G6PD deficiency are warranted in these high risk areas irrespective of gender for the prevention of disease states related to this condition.

## Introduction

Deficiency of G6PD enzyme affects more than 400 million people worldwide and is the most common enzymopathy in humans[1–3]. G6PD is a housekeeping enzyme that is vital for all cells and catalyses the generation of NADPH, which in turn enables cells to defend against triggers of oxidative stress[3]. Red blood cells are extremely vulnerable to oxidative stress, and in the absence of alternative sources of NADPH, makes them particularly dependent on G6PD for maintaining their integrity and function[4]. G6PD deficiency is an X-linked inherited disorder, whereby a higher proportion of males suffer from the deficiency than females[5].

Individuals with G6PD deficiency can present with a spectrum of disorders including acute massive haemolysis, neonatal jaundice, renal failure and chronic haemolytic anaemia induced by exposure to certain drugs, infections, fava beans, chemicals and herbal medicines[2,6]. The haemolytic episodes can vary from being asymptomatic and mild to severe and fatal in response to stressors[7]. The degree of enzyme deficiency (or variant), as well as the nature, dose and duration of exposure to the oxidative agent, together with host factors such as age, level of haemoglobin and concurrent infection will determine the severity of clinical manifestations[2,4]. While severe adverse events may require blood transfusions and renal dialysis, such procedures are expensive and not readily available in resource poor settings[7].

G6PD deficiency is important in malaria since it is believed to provide partial protection against the disease, thus increasingly selecting for these mutant genes[3,8–11]. The African variant G6PD A- is known to protect against severe malaria[12–14], while the Mediterranean (MED) and Asian variants are known to be more frequent in areas where *P*. *vivax* is endemic[15,16]. Leslie et al., in 2010[15] also demonstrated that G6PD deficiency MED variant conferred significant protection against vivax malaria infections. A recent study conducted by Dewasurendra et al[17], identified several G6PD gene variants and their association with malaria in a Sri Lankan population from Kataragama (Monaragala district, South-Eastern Sri Lanka). Nine of seventeen single nucleotide polymorphisms (SNPs) identified had a minor allele frequency greater than 10% and demonstrated a tendency for association with either disease severity or parasite density of uncomplicated malaria in males[17].

The dry zone of Sri Lanka has been endemic for malaria from historic times with epidemics occurring at periodic intervals[18–20], although at present (since 2012) there is no indigenous malaria transmission in Sri Lanka[21,22]. The districts of Anuradhapura (North Central Province) and Kurunegala (North Western Province) are two such previously high malaria endemic areas situated in the dry zone of Sri Lanka [22]. Since G6PD enzyme deficiency is known to offer protection against the disease, increased selection of the mutant gene in these two areas is to be expected. Since many disease states can be precipitated by G6PD enzyme deficiency, a high prevalence would be an important public health issue.

The current knowledge in Sri Lanka regarding the prevalence of G6PD deficiency is extremely sparse and gathered several decades previously[23–25]. Abeyaratne and Halpe (1968)[23], reported 4.86% of children (n = 432) developing acute intravascular haemolysis following treatment with primaquine in Anuradhapura; while Nagaratnam et al. (1969)[24] gave incidence rates of 1.26% and 1.28% for males and females respectively from Kegalle. Abeyaratne et al in 1976[25] also found a high frequency of G6PD deficiency in the North Central Province of Sri Lanka. The frequency was found to be higher in the ancient villages (7.1–20.9%) than in the recently colonized areas (3.5–3.7%), while it was 5.3% and 5.2% at the Anuradhapura hospital and school respectively. Another survey conducted throughout the island, except for eight districts in the Northern and Eastern Provinces, found an overall prevalence of < 3%[26].

The anti-malarial drugs primaquine, dapsone and the experimental drug tafenoquine are oxidative anti-malarials and can cause haemolytic anaemia in G6PD deficiency[5,27–29]. Currently, primaquine is the only approved anti-malarial that can be used to eliminate *Plasmodium vivax* hypnozoites and *Plasmodium falciparum* gametocytes, required for radical cure of patients with malaria[30]. And killing the reservoirs of parasites from within infected humans is an essential component for the successful elimination of malaria[31]. Thus, the occurrence of haemolysis due to administration of primaquine at certain dosing schedules in G6PD deficiency can undermine the implementation of malaria elimination programmes worldwide. As such, point of care G6PD testing to support safe treatment with primaquine for clearance of hypnozoites is recommended in those diagnosed with *P*. *vivax* infections, while it is no longer required for the single-dose transmission-blocking use in *P*. *falciparum*[30].

Several qualitative and quantitative methods are currently available for measuring the phenotypic G6PD enzyme status[7]. The fluorescent spot test which is the most widely used screening method provides only a qualitative result and requires an ultraviolet lamp and water bath[32]. Rapid and easy to use, point-of-care tests are the ideal for field settings. At present, there are two chromatographic rapid diagnostic test kits commercially available: BinaxNow G6PD (Alere Inc, USA) and the more recently developed CareStart G6PD (Access Bio, USA). The BinaxNow G6PD test requires an operating temperature of between 18–25°C and costs rather high, while CareStart G6PD appears to have overcome these constraints but remains a qualitative test able to detect enzyme deficiency at levels <2.7U/gHb[7]. The WST-8/1-methoxy PMS method (Dojindo Co., Japan), which has a similar test principle to the CareStart G6PD, has been adapted for use with dried blood spots[33] and enables both qualitative and quantitative measurements to be made[7,33,34].

Since the risk of haemolysis can be reduced by the use of extended dosing schedules of primaquine without compromising on efficacy[4], awareness regarding the prevalence of G6PD deficiency at individual or even community level would be of utmost importance. The objective of our study was to determine the extent of G6PD enzyme deficiency in selected persons attending the Teaching Hospitals of Anuradhapura and Kurunegala in Sri Lanka.

## Materials and Methods

### Sample collection

This was a descriptive, cross sectional study carried out at the Teaching Hospitals of Anuradhapura (North Central Province) and Kurunegala (North Western Province). These two areas were selected because they were highly endemic for malaria from historic times until the recent past and therefore expected to have a higher prevalence of G6PD deficient hosts. Ethical clearance for the study was obtained from the Ethics Review Committee of the Faculty of Medicine, University of Colombo (EC-12-134), as well as from the Teaching Hospital, Kurunegala. Permission for sample collection was obtained from the Director General of Health Services, Ministry of Health, Sri Lanka and the Directors of the respective hospitals. Participants were enrolled for the study after obtaining informed written consent.

Sample collection was carried out between November 2013 and June 2014 from persons attending the Teaching Hospitals at Anuradhapura and Kurunegala. A sample size of two thousand (one thousand from each district) was determined by assuming the prevalence of G6PD deficiency in the population as being 5% with an allowable error of 20% (precision = 0.01) at the 95% level of confidence. Patients presenting to the clinics at these two hospitals are usually referred to the ‘bleeding room’ for providing blood samples for any laboratory tests that may be required as part of their health screening/management process. We invited all patients presenting to the ‘bleeding room’ of each hospital during weekdays (morning and afternoon) to be part of the study. Blood samples were obtained from those who provided written consent until the required sample number was obtained. Two drops (~0.5mL) of blood from each person was collected on to 3MM filter papers (Whatman) and a simple form pasted on the zip-lock bag was completed giving the sample reference number with a code for each area given as a prefix, the date of collection, the address, age and gender of the participant. The blood spots were air dried and stored individually in zip-lock bags containing silica desiccant beads and refrigerated at +4°C until transported (in ice boxes) within 7 days to the Department of Parasitology, Faculty of Medicine, University of Colombo where the laboratory investigations were conducted.

### G6PD functional assay

The WST-8/1-methoxy PMS method described initially by Tantular and Kawamoto[34] utilizes WST-8, a tetrazolium salt which does not react with haemoglobin, and a less light-sensitive form of the phenazine methosulphate (PMS) hydrogen carrier called 1-methoxyPMS, which in the presence of NADPH converts WST-8 to formazan (orange colour). The strong orange colour of the WST-8 formazan formed in normal blood samples can be distinguished with the naked eye (qualitative) or by reading the absorbance (quantitative) using an ELISA microplate reader.

The method was modified by Kuwahata et al.[33] by optimizing to a 96-well plate format using dried bloodspots with internal standards as controls and validated for mass screening to determine the prevalence of G6PD deficiency[33,35]. The reactive intensity and retentive ability for G6PD activity in blood samples spotted onto filter papers is known to remain up to 10–14 days at 4°C[33,35,36]. The qualitative performance of the WST-8/1-methoxy PMS method evaluated recently against spectrophotometry (Trinity Biotech, Ireland) showed sensitivity and specificity rates of 88.9% (95% CI 73.9–96.9)and 95.6% (95% CI 93.6–97.1) respectively at the 10% enzyme activity level and 84.2% (95% CI 72.1–92.5) and 98.2% (95% CI 96.8–99.1) at the 30% level[37].

**Fig 1:**
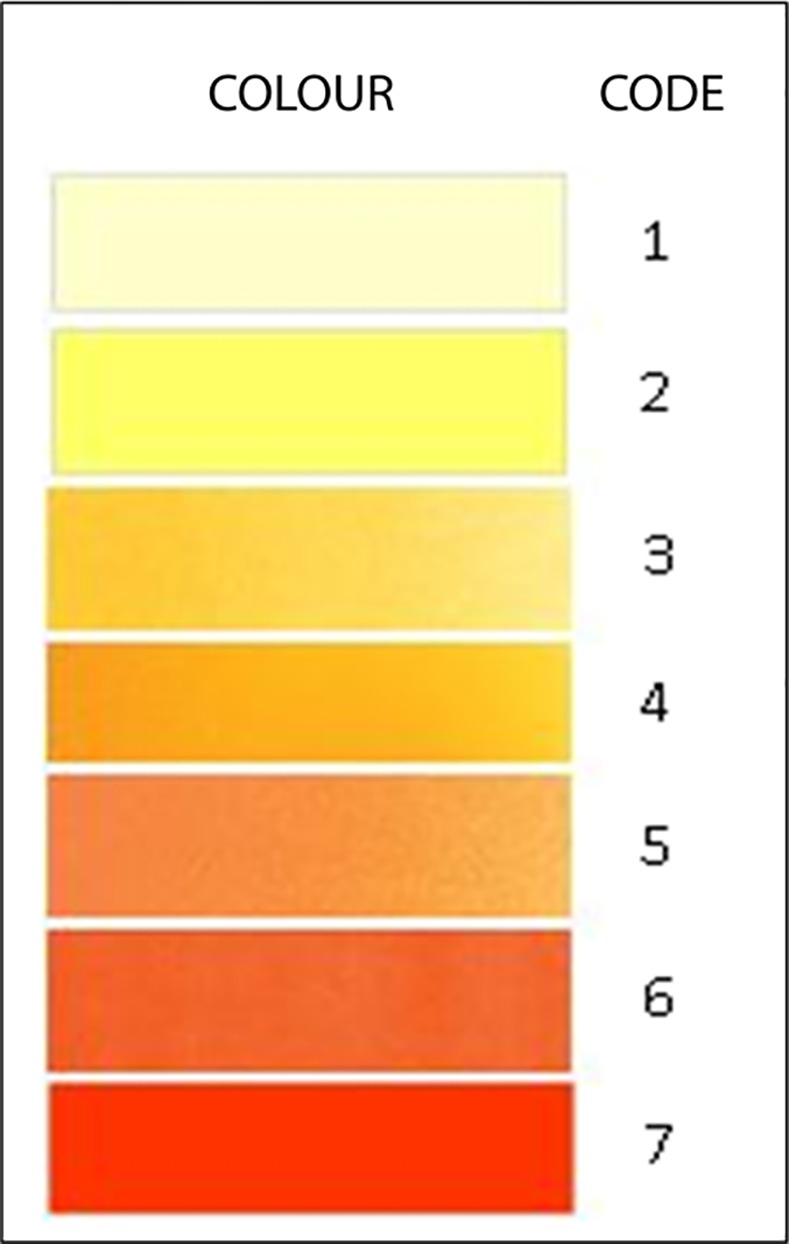
Colour Chart.

The G6PD enzyme activity detection assay for our study was established based on the previously validated modified WST-8/1-methoxy PMS method described by Kuwahata et al. (2010)[33]. The WST-8/1-methoxyPMS mixture was purchased from Dojindo Laboratories (Japan), while the commercial standard reagent of known normal G6PD activity (G6PD control normal, Trinity Biotech, Ireland) was used to create the panel of internal controls for each assay (normal, moderately deficient: ~30% enzyme activity, severely deficient: ~10% enzyme activity). Plates were incubated for 2h at ambient temperature (~30°C), and were then inspected visually by two separate observers with the aid of a colour chart (Fig 1) and colour codes were recorded. The observers were blinded to each other’s interpretations and to the test outcomes. For quantitative analysis, the optical density was quantified in a microplate reader at OD^450-630^nm.

For further validation of our assay, 2mL of blood was collected into anticoagulant containing blood tubes from 20 persons (10 from each hospital) and tested with a commercially available kinetic UV determination test kit for G6PD (manufactured by Biochemical Enterprise—BEN S. r. l, Italy, available at http://www.bensrl.it/) from a laboratory at a private hospital in Colombo. The number so tested was limited due to the necessity of having to provide 2mL of whole blood and the high cost involved per test.

### Data analysis

G6PD levels were determined in reference to the control panels (for the WST-8/1-methoxy PMS method) and normal reference range (for the commercial test kit). Prevalence of G6PD deficiency was calculated as a percentage of the total number of positive persons from the total number tested for each district and according to gender. Chi square test was used to determine any significant difference in gender distribution of enzyme deficiency and for comparative analysis between sites. The frequencies of distribution of enzyme activity levels were determined as percentages for each gender. Enzyme concentrations were calculated as a percentage to the normal reference control using MS Excel 2007. Frequencies of colour code observations as well as discrepancies between observers for varying enzyme activity levels were calculated as percentages for each category.

## Results

### Prevalence of G6PD enzyme deficiency

A total of 2059 filter paper blood samples were collected (1018 from TH Anuradhapura, S1 File; 1041 from TH Kurunegala, S2 File). Almost 70% of samples were from females (705 from Anuradhapura; 728 from Kurunegala). The rate of G6PD deficiency in the sample was found to be 13.95% (n = 142) and 7.97% (n = 83) in the Teaching Hospitals of Anuradhapura and Kurunegala respectively (Table 1). The G6PD prevalence was significantly greater in Anuradhapura when compared to Kurunegala (P<0.0001). Surprisingly, the prevalence of enzyme deficiency among females was similar to that of males in each district (P>0.05; Table 2). When compared between districts, the number of females with G6PD deficiency was significantly greater in Anuradhapura (total, P<0.001; <10%, P = 0.04; 10–30%, P<0.001) while no such difference was observed between the males (P>0.05).

**Table 1:** Prevalence of G6PD deficiency in the two Teaching Hospitals (n = 2059).

	Total (≤ 30%)			<10%			10–30%		
	%	n^a^	95% CI^b^	%	n^a^	95% CI^b^	%	n^a^	95% CI^b^
TH Anuradhapura (n^a^ = 1018) [95% CI^b^: 54.53–58.67]	13.95	142	14.68–22.72	2.75	28	3.58–8.82	11.20	114	19.17–24.07
TH Kurunegala (n^a^ = 1041) [95% CI^b^: 55.19–58.95]	7.97	83	11.0–19.94	1.63	17	1.87–8.33	6.34	66	14.89–21.39
X^2^	18.88			3.01			15.23		
P value	<0.001			0.083			<0.001		

^a^n denotes the number of samples.

^b^95% CI denotes the interval within which lies the mean percentage level of G6PD enzyme in the population predicted at a 95% level of confidence.

**Table 2:** Prevalence of G6PD deficiency according to gender.

	Severity	Number	Male	Female^a^	X^2^	P value
**TH Anuradhapura** (Males: n^b^ = 313; 95% CI^c^: 56.99–64.89) (Females: n^b^ = 705; 95% CI^c^: 52.20–57.02)	≤10%	28	7 (2.24%)	21 (2.98%)	0.45	0.504
	>10–30%	114	28 (8.95%)	86 (12.20%)	2.31	0.129
	Total	142	35 (11.18%) 95% CI^c^: 11.25–24.91	107 (15.18%) 95% CI^c^: 14.78–22.72	2.88	0.089
**TH Kurunegala** (Males: n^b^ = 313; 95% CI^c^: 55.82–63.02) (Females: n^b^ = 728; 95% CI^c^: 53.85–58.25)	≤10%	17	7 (2.24%)	10 (1.37%)	1.01	0.314
	>10–30%	66	18 (5.75%)	48 (6.59%)	0.26	0.609
	Total	83	25 (7.99%) 95% CI^c^: 7.20–22.96	58 (7.97%) 95% CI^c^: 10.29–20.99	<0.01	0.991

^a^Prevalence of G6PD deficiency was significantly greater among females in Anuradhapura than in Kurunegala (P<0.05).

^b^n denotes the number of samples.

^c^95% CI denotes the interval within which lies the mean percentage level of G6PD enzyme in the population predicted at a 95% level of confidence.

**Fig 2:**
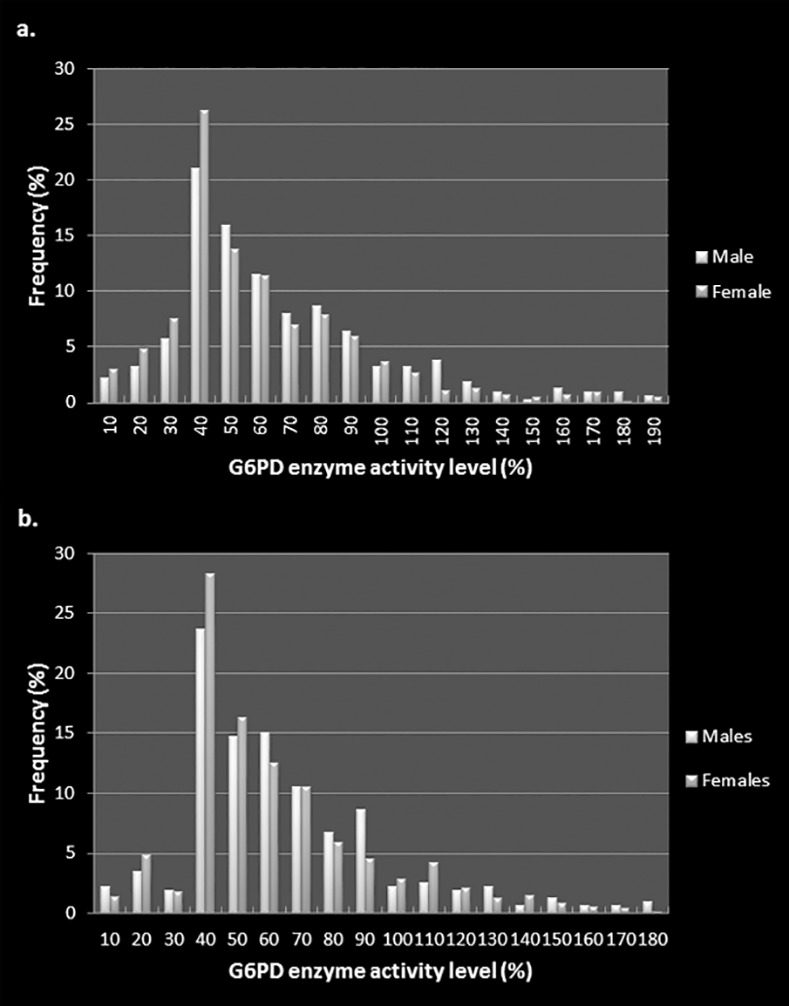
Distribution of G6PD enzyme activity in the Anuradhapura district (a) and b. in the Kurunegala district (b).

The distribution of enzyme activity levels was similar in the two districts with a majority of persons having levels between 30–60% of the normal control (Fig 2A and 2B). The mean enzyme concentrations were 60.98% and 59.42% for males and 54.61% and 56.05% for females in Anuradhapura and Kurunegala districts respectively. The ages of those with enzyme deficiency (<30% activity) ranged from 2 months to 85 years (median 50) in Anuradhapura while it was 25 to 83 years in Kurunegala (median 60). The majority of persons sampled from each district were aged between 46 and 65 years (Table 3). Test results from the commercial test kit were comparable to our assay, with both tests done on 20 persons (10 from each district) identifying enzyme deficiency in one person from Kurunegala while the rest had normal levels.

**Table 3:** Distribution of G6PD enzyme activity according to age.

	G6PD activity	Number	Age (years)			
			<25	25–45	46–65	>65
**TH Anuradhapura**	≤10%	28	5	4	13	6
	>10–30%	114	23	22	56	13
	>30–60%	515	110	127	231	47
	>60%	361	80	104	139	38
	Total	1018	218 (21.4%)	257 (25.3%)	439 (43.1%)	104 (10.2%)
**TH Kurunegala**	≤10%	17	-	1	11	5
	>10–30%	66	-	7	42	17
	>30–60%	582	27	85	337	133
	>60%	376	25	75	211	65
	Total	1041	52 (5.0%)	168 (16.1%)	601 (57.7%)	220(21.1%)

### Qualitative Vs Quantitative assessment

**Fig 3:**
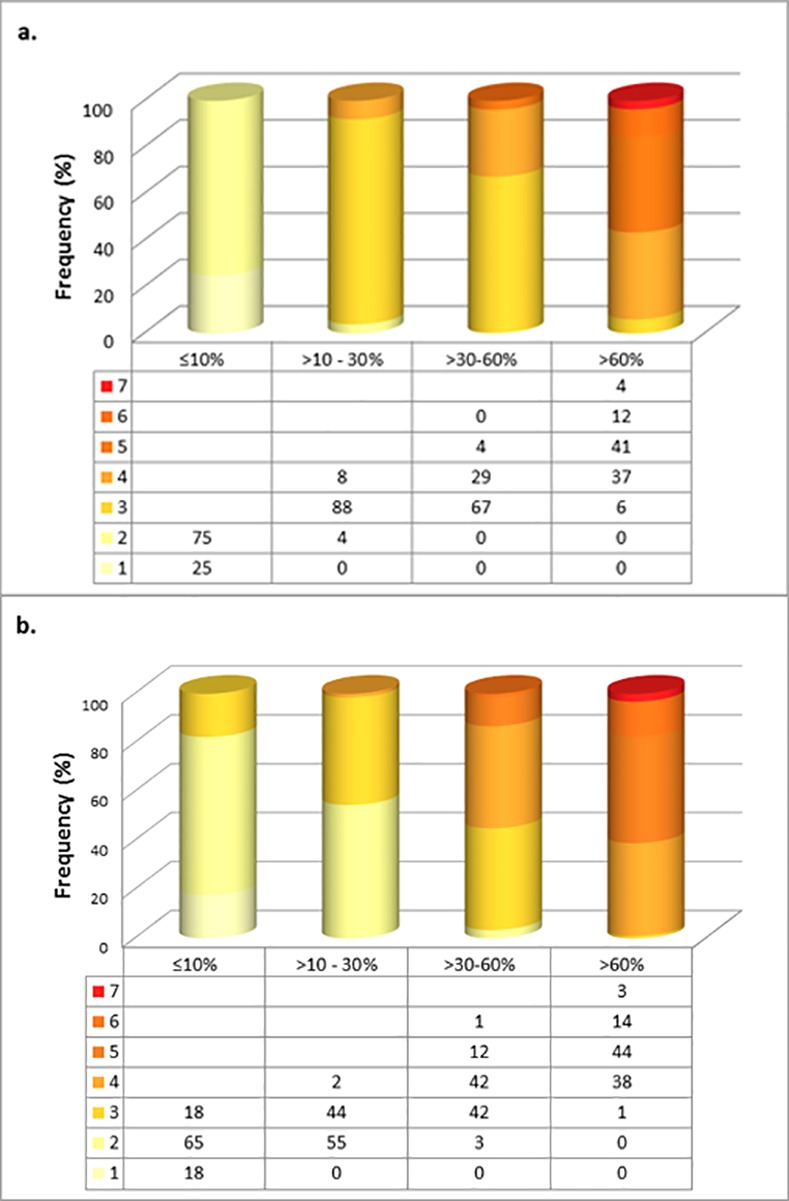
Colour code observations at varying G6PD activity levels in samples from Anuradhapura district (a) and Kurunegala district (b).

**Fig 4:**
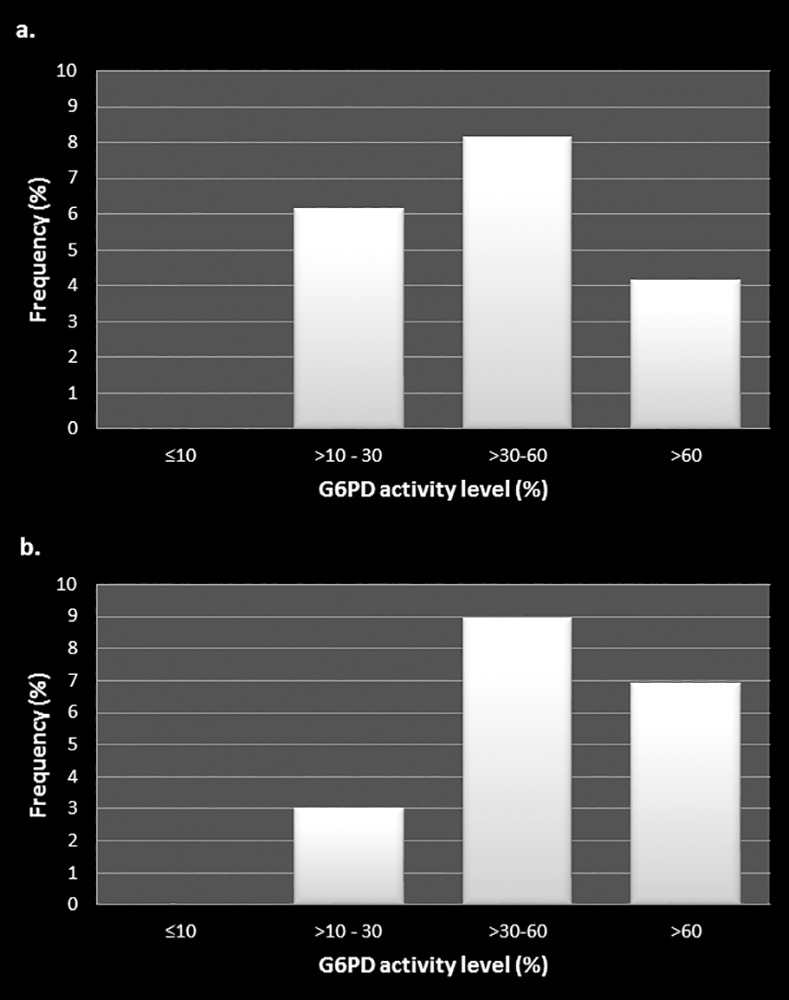
Colour code discrepancies between observers at varying G6PD activity levels in samples from Anuradhapura district (a) and Kurunegala district (b).

The frequencies of colour code observations for varying enzyme activity levels are shown separately for each district in Fig 3A and 3B. Colour codes 1 and 2 correspond with moderate to severe enzyme deficiency states (<30% activity) while codes 5 through 7 appear to relate with normal enzyme levels. Discrepancies between observers in coding of colours was nil in severe deficiency (<10% activity) and less than 10% at all other levels of enzyme activity as revealed in Fig 4A and 4B. The majority of colour coding discrepancies for each district occurred in persons aged between 46 and 65 years and among females (Table 4).

**Table 4:** Distribution of colour code discrepancies according to age and gender.

	G6PD activity	Number	Age (years)				Gender		
			<25	25–45	46–65	>65	Male	Female	
**TH Anuradhapura**	≤10%	-	-	-	-	-	-		-
	>10–30%	7	2	2	3	-	3		4
	>30–60%	42	10	7	21	4	10		32
	>60%	15	3	7	5	-	4		11
	Total	64	15 (23.4%)	16 (25.0%)	29 (45.3%)	4 (6.3%)	17 (26.6%)		47 (73.4%)
**TH Kurunegala**	≤10%	-	-	-	-	-	-		-
	>10–30%	2	-	-	1	1	1		1
	>30–60%	52	2	6	25	19	14		38
	>60%	26	-	7	15	4	12		14
	Total	80	2 (2.5%)	13 (16.3%)	41 (51.3%)	24 (30.0%)	27 (33.8%)		53 (66.3%)

## Discussion

The World Health Organization has mapped the frequency of G6PD deficiency throughout the world with the highest rates of up to 26% of the general population in Central Africa and the Middle East and the lowest <0.5% in the Northern Hemisphere including Northern Europe, United Kingdom, the Scandinavian countries, Russia and northern China[3]. A more recent survey based on a geo-statistical model-based map across malaria endemic countries predicted the highest median G6PD deficiency prevalence (peaking at 32.5%) across sub-Saharan Africa and the Arabian Peninsula, with a generally lower prevalence across central and southeast Asia, rarely exceeding 20%, although the majority of G6PD deficient individuals (67.5% median estimate) were from Asian countries[38].

The gene encoding G6PD maps to the q28 locus on the long arm of the X chromosome and spans 18 kb and consists of 13 exons[39]. G6PD deficiency is genetically heterogeneous with 186 different allelic mutations in the G6PD gene that have been identified[40,41], generating more than 400 different enzyme variants as reported on the basis of diverse biochemical characteristics[42]. The different polymorphic mutants that underlie G6PD deficiency in various parts of the world explains to a great extent the diversity of occurrence in clinical manifestations associated with this condition[40]. Since all identified mutations are in the coding regions of the G6PD gene, the reduction in enzyme activity is believed to be associated with instability of the enzyme rather than impaired gene expression[4].

The WHO (1989)[3] classifies G6PD deficiency according to the level of G6PD enzyme activity with Class I being associated with chronic non-spherocytic haemolytic anaemia (CNSHA); Class II as severely deficient with less than 10% residual activity; Class III being moderately deficient with 10–60% residual activity; Class IV showing normal activity of 60–150%; and Class V being increased activity. Adverse events are generally confined to individuals with G6PD activity < 10% of normal (WHO Class II)[3]. The common African variant G6PD A- usually causes a mild to moderate deficiency while the MED variant is more severe[5].

In addition to anti malarial drugs, many analgesics, antibacterials as well as other miscellaneous drugs too can induce acute haemolysis in susceptible individuals[6]. Dapsone is one of the drugs in the multi-drug regimen used for treating leprosy and is administered as a daily dose varying from 6 months (paucibacillary forms of leprosy) to one year duration (multi-bacillary forms of leprosy) depending on the type of leprosy[43]. Dapsone is also used in the treatment of many dermatological conditions while its anti-inflammatory and antiprotozoal effects are useful against numerous other conditions as well[44]. A case of Dapsone induced haemolytic anaemia was reported from the Teaching Hospital, Anuradhapura, in a boy treated for leprosy and having normal G6PD activity[45], while a case of nitrofurantoin induced haemolysis was recently reported in a G6PD deficient pregnant woman[46].

Haemolytic anaemias have also been associated with G6PD deficiency and fava beans (*Vicia faba*, the Broad Bean), a species of bean native to North Africa and Southwest Asia, and extensively cultivated elsewhere[47]. No cases of favism have been reported in Sri Lanka to date, probably because the common type of bean used for consumption in Sri Lanka is *Phaseolus vulgaris* L. Furthermore, chemicals such as naphthalene and methylene blue, certain antifungals, henna and infectious diseases caused by viruses and bacteria have all been associated with the occurrence of haemolytic anaemias in G6PD deficient individuals[6].

Our data shows an overall prevalence rate of almost 14% and 8% in Anuradhapura and Kurunegala areas respectively, while rates for severe deficiency (<10% enzyme activity) are under 3%. A recent median predicted allele frequency map of G6PD deficiency across malaria endemic countries predicted allele frequencies of up to 15% within Sri Lanka, especially in the northern, north western, north central and eastern parts of the country[38]. In Sri Lanka, especially in Anuradhapura and Kurunegala, malaria has been a life threatening occurrence for centuries [18–20,22], and it is to be expected that normal individuals would be selected against by malaria and G6PD deficient individuals would be favoured. Heterozygous females would have the greatest selective advantage since they would not suffer from favism or other G6PD associated outcomes while being protected against severe malaria.

The X-linked inheritance of the G6PD gene makes hemizygous males vulnerable to clinical manifestations in the presence of a variant, while deficiency in females is more complex[6]. Although females inherit 2 copies of the X chromosome, one of them is inactivated early in embryogenesis in a random process of Lyonization[48]. Homozygous females will thus be either normal or deficient for G6PD depending on the type of allele they possess, akin to hemizygous males[4]. However, heterozygous females will be mosaic and express two red cell populations consisting of normal and deficient cells[49]. The proportions of these two red cell populations can be variable depending on lyonization, with unfavourable lyonization (inactivation of the normal gene) giving rise to more deficient than normal red cells[50]. In the presence of life threatening malaria, unfavourable lyonization may have provided an advantage for survival, thus selecting for such heterozygous females as revealed by the high numbers of G6PD deficient females observed in our study. Comparative genotyping of 12 SNP markers between one hundred G6PD deficient and a similar number of normal individuals is currently underway. Since G6PD deficient individuals are susceptible to potentially severe and life-threatening haemolysis, implementation of screening tests and educational programmes are warranted in these high risk areas irrespective of gender.

At present, the Brewer’s test[51] is used in government hospitals in Sri Lanka for determining the risk of haemolysis. This is a non-specific screening test for G6PD deficiency and shown to be affected by different haemoglobinopathies[52]. According to hospital records for 2016, TH Anuradhapura had screened 89 patients and 4 were G6PD deficient (4.49%); In TH Kurunegala, 74 patients had been tested and 15 were found to be deficient (20.27%). The WST-8/1-methoxyPMS method is an effective screening method that could be established in government hospitals, and would be useful for detection of at least severe G6PD deficiency (<10%) just by observation with the naked eye. However, the test requires importation of reagents and strict maintenance of the cold chain with sample processing done as early as possible for best results.

## Conclusions

We conclude that screening and educational programmes for G6PD deficiency are warranted in these high risk areas irrespective of gender in order to prevent the occurrence of disease states related to G6PD deficiency.

## Supporting Information

S1 File (PDF)

S2 File (PDF)
