# Supplementary material for: Prevalence of G6PD deficiency in selected populations from two previously high malaria endemic areas of Sri Lanka
Source: PLoS One. 2017 Feb 2;12(2):e0171208. doi: 10.1371/journal.pone.0171208 (PMC5289554; doi:10.1371/journal.pone.0171208)
Supplement: S1 File — (PDF) [file pone.0171208.s001.pdf]

Plate 1								
**Sample**	**450nm**	**630nm**	**450-630nm**	**%**	**Colour O1**	**Colour O2**	**Age**	**Sex**
Blank	**0**	**0**	**0**		**No**	**No**		
Inactivated	**0.2**	**0.118**	**0.082**		**No**	**No**		
Sub negative	**0.022**	**0.013**	**0.009**		**No**	**No**		
100%	**0.535**	**0.082**	**0.453**		**5**	**5**		
60%	**0.421**	**0.082**	**0.339**		**4**	**4**		
30%	**0.329**	**0.083**	**0.246**		**3**	**3**		
10%	**0.197**	**0.054**	**0.143**		**2**	**2**		
A1	0.265	0.078	**0.187**	18.2	3	3	41	F
A2	0.387	0.154	**0.233**	31.7	3	3	2	M
A3	0.556	0.099	**0.457**	97.5	4	4	28	F
A4	0.587	0.104	**0.483**	105.2	4	4	37	F
A5	0.469	0.137	**0.332**	60.8	4	4	37	F
A6	0.466	0.177	**0.289**	48.2	3	3	60	F
A7	0.429	0.129	**0.3**	51.4	3	3	30	M
A8	0.272	0.101	**0.171**	13.5	3	3	60	F
A9	0.39	0.104	**0.286**	47.3	3	3	55	F
A10	0.393	0.098	**0.295**	49.9	3	3	75	F
A11	0.686	0.154	**0.532**	119.6	5	5	45	M
A12	0.409	0.112	**0.297**	50.5	3	3	54	F
A13	0.203	0.014	**0.189**	18.8	3	3	53	F
A14	0.36	0.109	**0.251**	37.0	3	3	46	F
A15	0.568	0.262	**0.306**	53.2	3	3	39	M
A16	0.99	0.316	**0.674**	161.3	5	5	35	F
A17	0.317	0.096	**0.221**	28.2	3	3	63	F
A18	0.623	0.164	**0.459**	98.1	4	4	59	F
A19	0.452	0.051	**0.401**	81.1	4	4	76	F
A20	0.524	0.07	**0.454**	96.7	4	4	75	F
A21	0.396	0.027	**0.369**	71.7	4	4	45	F
A22	0.369	0.113	**0.256**	38.5	3	3	58	M
A23	0.711	0.185	**0.526**	117.8	4	4	74	F
A24	0.291	0.079	**0.212**	25.5	3	3	64	F
A25	0.907	0.138	**0.769**	189.2	6	6	21	F
A26	0.509	0.146	**0.363**	69.9	4	4	55	F
A27	0.786	0.521	**0.265**	41.1	3	3	52	M
A28	0.672	0.132	**0.54**	121.9	5	5	50	F
A29	0.891	0.546	**0.345**	64.6	4	4	4	F
A30	0.367	0.07	**0.297**	50.5	3	3	43	M
A31	0.434	0.136	**0.298**	50.8	3	3	43	F
A32	0.369	0.051	**0.318**	56.7	3	3	38	F
A33	0.399	0.067	**0.332**	60.8	4	4	66	F
A34	0.29	0.115	**0.175**	14.7	3	3	46	M
A35	0.493	0.077	**0.416**	85.5	5	5	49	M
A36	0.227	0.056	**0.171**	13.5	3	3	69	F
A37	0.358	0.058	**0.3**	51.4	3	3	56	F
A38	0.346	0.093	**0.253**	37.6	3	3	37	F
A39	0.281	0.059	**0.222**	28.5	3	3	38	F
A40	0.335	0.053	**0.282**	46.1	3	3	63	F
A41	0.393	0.073	**0.32**	57.3	3	3	52	F
A42	0.412	0.131	**0.281**	45.8	3	3	11m	M
A43	0.181	0.052	**0.129**	1.1	2	2	62	M
A44	0.274	0.076	**0.198**	21.4	3	3	45	F
A45	0.265	0.057	**0.208**	24.4	3	3	67	F
A46	0.454	0.108	**0.346**	64.9	4	4	50	F
A47	0.242	0.067	**0.175**	14.7	3	3	77	M
A48	0.259	0.055	**0.204**	23.2	3	3	38	M
A49	0.456	0.069	**0.387**	77.0	4	4	35	F
A50	0.387	0.119	**0.268**	42.0	3	3	51	F
A51	0.302	0.081	**0.221**	28.2	3	3	52	F
A52	0.376	0.046	**0.33**	60.2	4	4	40	M
A53	0.397	0.032	**0.365**	70.5	4	4	50	F
A54	0.381	0.065	**0.316**	56.1	4	4	20	F
A55	0.192	0.036	**0.156**	9.1	2	2	67	F
A56	0.753	0.055	**0.698**	168.4	6	6	37	F
A57	0.618	0.08	**0.538**	121.3	5	5	33	F
A58	0.506	0.125	**0.381**	75.2	4	4	25	M
A59	0.424	0.083	**0.341**	63.4	4	4	76	F

A60	0.412	0.087	**0.325**	58.7	3	3	69	F
A61	0.414	0.083	**0.331**	60.5	4	4	65	F
A62	0.411	0.12	**0.291**	48.8	3	3	55	M
A63	0.316	0.093	**0.223**	28.8	3	3	65	F
A64	0.353	0.129	**0.224**	29.1	3	3	60	F
A65	0.254	0.085	**0.169**	12.9	3	3	58	F
A66	0.385	0.102	**0.283**	46.4	3	3	47	F
A67	0.29	0.071	**0.219**	27.6	3	3	61	F
A68	0.407	0.094	**0.313**	55.2	3	3	49	F
A69	0.741	0.062	**0.679**	162.8	6	6	47	M
A70	0.275	0.09	**0.185**	17.6	3	3	2\12	M
A71	0.434	0.139	**0.295**	49.9	3	3	68	F
A72	0.239	0.041	**0.198**	21.4	3	3	26	F
A73	0.448	0.116	**0.332**	60.8	4	4	4	F
A74	0.692	0.102	**0.59**	136.6	4	4	46	F
A75	0.483	0.225	**0.258**	39.1	3	3	66	M
A76	0.493	0.084	**0.409**	83.4	5	5	23	F
A77	0.416	0.176	**0.24**	33.8	3	3	2y6m	F
A78	0.263	0.1	**0.163**	11.1	3	3	64	F
A79	0.77	0.109	**0.661**	157.5	5	5	33	F
A80	0.54	0.298	**0.242**	34.4	3	3	63	F
A81	0.638	0.155	**0.483**	105.2	5	5	60	F
A82	0.506	0.101	**0.405**	82.3	5	5	66	F
A83	0.291	0.076	**0.215**	26.4	3	3	71	M
A84	0.199	0.073	**0.126**	0.3	1	1	49	F
A85	0.196	0.068	**0.128**	0.9	1	1	11	M
A86	0.461	0.07	**0.391**	78.1	4	4	21	F
A87	0.411	0.111	**0.3**	51.4	3	3	10	F
**Plate 2**								
**Sample**	**450nm**	**630nm**	**450-630nm**	**%**	**Colour O1**	**Colour O2**	**Age**	**Sex**
Blank	**0**	**0**	**0**		**No**	**No**		
Inactivated	**0.293**	**0.108**	**0.185**		**No**	**No**		
sub negative	**0.004**	**0**	**0.004**		**No**	**No**		
100%	**0.588**	**0.083**	**0.505**		**5**	**5**		
60%	**0.437**	**0.084**	**0.353**		**4**	**4**		
30%	**0.335**	**0.076**	**0.259**		**3**	**3**		
10%	**0.247**	**0.08**	**0.167**		**2**	**2**		
A88	0.346	0.169	**0.177**	11.0	3	3	47	F
A89	0.795	0.1	**0.695**	151.3	5	5	48	M
A90	0.53	0.172	**0.358**	60.0	4	4	65	M
A91	0.353	0.017	**0.336**	54.1	4	4	66	F
A92	0.283	0.088	**0.195**	15.7	3	3	48	F
A93	1.061	0.307	**0.754**	167.3	6	6	56	F
A94	0.346	0.053	**0.293**	42.4	3	3	46	F
A95	0.343	0.108	**0.235**	26.7	3	3	36	F
A96	0.454	0.175	**0.279**	38.6	3	3	1	F
A97	0.277	0.062	**0.215**	21.3	3	3	19	F
A98	0.674	0.373	**0.301**	44.6	3	3	35	M
A99	0.462	0.118	**0.344**	56.2	3	3	32	F
A100	0.763	0.077	**0.686**	148.9	5	5	55	F
A101	0.315	0.096	**0.219**	22.4	3	3	59	F
A102	0.81	0.052	**0.758**	168.4	6	6	32	F
A103	0.32	0.052	**0.268**	35.6	3	3	22	F
A104	0.556	0.116	**0.44**	82.2	4	4	11	F
A105	0.579	0.142	**0.437**	81.4	4	4	48	M
A106	0.352	0.092	**0.26**	33.5	3	3	45	F
A107	0.428	0.049	**0.379**	65.7	4	4	2	F
A108	0.609	0.031	**0.578**	119.6	5	5	22	M
A109	0.561	0.054	**0.507**	100.4	4	4	17	M
A110	0.399	0.031	**0.368**	62.7	4	4	58	F
A111	0.512	0.068	**0.444**	83.3	4	4	17	F
A112	0.486	0.132	**0.354**	58.9	4	4	40	M
A113	0.35	0.076	**0.274**	37.3	3	3	48	F
A114	0.712	0.109	**0.603**	126.4	5	5	57	M
A115	0.452	0.052	**0.4**	71.4	3	4	53	M
A116	0.279	0.08	**0.199**	16.9	3	3	50	F
A117	0.732	0.447	**0.285**	40.2	3	3	51	F

A118	0.505	0.046	**0.459**	87.4	4	4	52	F
A119	0.316	0.075	**0.241**	28.3	3	3	22	F
A120	0.422	0.131	**0.291**	41.9	3	3	53	F
A121	0.376	0.074	**0.302**	44.9	3	3	22	M
A122	0.437	0.093	**0.344**	56.2	3	3	48	F
A123	0.394	0.063	**0.331**	52.7	3	3	35	F
A124	0.55	0.103	**0.447**	84.1	4	4	32	M
A125	0.691	0.428	**0.263**	34.3	3	3	4	F
A126	0.218	0.06	**0.158**	5.8	2	2	67	F
A127	0.322	0.089	**0.233**	26.2	3	3	35	F
A128	0.582	0.145	**0.437**	81.4	4	4	6	F
A129	0.302	0.114	**0.188**	14.0	3	3	30	F
A130	0.545	0.08	**0.465**	89.0	4	4	23	M
A131	0.508	0.102	**0.406**	73.0	4	4	36	F
A132	0.289	0.062	**0.227**	24.5	3	3	43	F
A133	0.393	0.058	**0.335**	53.8	3	3	73	F
A134	0.773	0.053	**0.72**	158.1	6	6	68	F
A135	0.334	0.092	**0.242**	28.6	3	3	61	M
A136	0.541	0.214	**0.327**	51.6	4	4	62	M
A137	0.595	0.312	**0.283**	39.7	3	3	48	F
A138	0.537	0.105	**0.432**	80.1	4	4	14	F
A139	0.202	0.038	**0.164**	7.5	2	2	48	F
A140	0.17	0.029	**0.141**	1.2	2	2	64	M
A141	0.39	0.037	**0.353**	58.7	4	4	57	F
A142	0.158	0.019	**0.139**	0.7	1	1	51	M
A143	0.738	0.349	**0.389**	68.4	4	4	37	M
A144	0.527	0.211	**0.316**	48.6	4	4	45	F
A145	0.378	0.091	**0.287**	40.8	3	3	53	F
A146	0.609	0.205	**0.404**	72.5	4	4	48	M
A147	0.496	0.21	**0.286**	40.5	3	3	39	F
A148	0.357	0.074	**0.283**	39.7	3	3	34	F
A149	0.437	0.086	**0.351**	58.1	4	4	29	M
A150	0.677	0.062	**0.615**	129.7	5	5	32	F
A151	0.422	0.14	**0.282**	39.4	3	3	35	F
A152	0.266	0.089	**0.177**	11.0	3	3	60	M
A153	0.328	0.117	**0.211**	20.2	3	3	66	F
A154	0.47	0.162	**0.308**	46.5	3	3	56	F
A155	0.433	0.092	**0.341**	55.4	4	4	57	M
A156	0.42	0.154	**0.266**	35.1	3	3	61	F
A157	0.76	0.08	**0.68**	147.3	5	5	32	F
A158	0.961	0.361	**0.6**	125.6	4	5	4	M
A159	0.589	0.304	**0.285**	40.2	3	3	42	F
A160	0.561	0.157	**0.404**	72.5	4	4	65	F
A161	0.365	0.109	**0.256**	32.4	3	3	32	M
A162	0.972	0.273	**0.699**	152.4	5	5	1y6m	F
A163	0.379	0.127	**0.252**	31.3	3	3	60	F
A164	0.674	0.1	**0.574**	118.6	4	4	2y3m	M
A165	0.861	0.1	**0.761**	169.2	6	6	42	F
A166	1.01	0.205	**0.805**	181.2	5	6	36	F
A167	0.239	0.077	**0.162**	6.9	2	2	70	F
A168	0.864	0.232	**0.632**	134.3	5	5	34	M
A169	0.412	0.122	**0.29**	41.6	3	3	52	F
A170	0.334	0.131	**0.203**	18.0	3	3	66	F
A171	0.258	0.081	**0.177**	11.0	3	3	9\12	F
A172	0.365	0.089	**0.276**	37.8	3	3	16	F
A173	0.21	0.058	**0.152**	4.2	2	2	33	F
A174	0.256	0.079	**0.177**	11.0	3	3	60	F
**Plate 3**								
**Sample**	**450nm**	**630nm**	**450-630nm**	**%**	**Colour O1**	**Colour O2**	**Age**	**Sex**
Blank	**0**	**0**	**0**		**No**	**No**		
Inactivated	**0.246**	**0.118**	**0.128**		**No**	**No**		
Sub negative	**0.006**	**0.003**	**0.003**		**No**	**No**		
100%	**0.609**	**0.087**	**0.522**		**5**	**5**		
60%	**0.522**	**0.096**	**0.426**		**4**	**4**		
30%	**0.325**	**0.012**	**0.313**		**3**	**3**		
10%	**0.285**	**0.043**	**0.242**		**2**	**2**		
A175	0.482	0.118	**0.364**	46.3	4	4	23	F

A176	0.411	0.086	0.325	34.0	3	3	33	F
A177	0.241	0.004	0.237	6.3	2	2	43	M
A178	0.273	0.043	0.23	4.1	2	2	3 4\12	F
A179	0.735	0.213	0.522	96.1	5	5	9	M
A180	0.325	0.071	0.254	11.6	3	3	21	M
A181	0.337	0.021	0.316	31.2	3	3	33	F
A182	0.371	0.032	0.339	38.4	3	3	45	F
A183	0.353	0.031	0.322	33.1	3	3	57	M
A184	0.229	0.01	0.219	0.6	1	1	73	F
A185	0.342	0.019	0.323	33.4	3	3	38	F
A186	0.591	0.074	0.517	94.5	5	5	63	F
A187	0.415	0.079	0.336	37.5	4	4	55	M
A188	0.302	0.026	0.276	18.6	3	3	42	F
**Plate 4**								
**Sample**	**450nm**	**630nm**	**450-630nm**	**%**	**Colour O1**	**Colour O2**	**Age**	**Sex**
Blank	0	0	0		No	No		
Inactivated	0.139	0.089	0.05		No	No		
Sub negative	0.777	0.684	0.093		No	No		
100%	0.592	0.066	0.526		5	5		
60%	0.29	0.066	0.224		3	3		
30%	0.208	0.087	0.121		2	2		
10%	0.163	0.074	0.089		2	2		
A189	0.334	0.056	0.278	57.2	3	3	9months	F
A190	0.32	0.088	0.232	48.5	3	3	16	F
A191	0.353	0.052	0.301	61.6	4	4	67	F
A192	0.361	0.079	0.282	58.0	3	3	8	F
A193	0.808	0.529	0.279	57.4	5	5	28	F
A194	0.275	0.086	0.189	40.3	3	3	61	F
A195	0.498	0.078	0.42	84.1	4	4	34	F
A196	0.849	0.488	0.361	72.9	6	6	40	F
A197	0.437	0.07	0.367	74.1	4	4	41	F
A198	1.103	0.295	0.808	157.7	6	6	44	M
A199	0.259	0.122	0.137	30.5	3	3	58	F
A200	0.231	0.067	0.164	35.6	3	3	45	M
A201	0.242	0.035	0.207	43.7	3	4	46	F
A202	0.23	0.042	0.188	40.1	3	3	29	F
A203	0.222	0.067	0.155	33.9	3	3	4months	F
A204	0.261	0.055	0.206	43.6	4	4	60	F
A205	0.275	0.09	0.185	39.6	3	3	2	F
A206	0.353	0.031	0.322	65.5	4	4	6	F
A207	0.295	0.13	0.165	35.8	3	3	yrn 10mnth	F
A208	0.561	0.072	0.489	97.2	6	6	35	M
A209	0.339	0.073	0.266	54.9	3	3	31	F
A210	0.392	0.121	0.271	55.9	3	3	54	F
A211	0.939	0.074	0.865	168.5	7	7	60	M
A212	0.486	0.094	0.392	78.8	4	4	58	F
A213	0.361	0.037	0.324	65.9	3	4	17	F
A214	0.336	0.056	0.28	57.6	3	3	36	F
A215	0.461	0.047	0.414	83.0	4	4	40	F
A216	0.278	0.038	0.24	50.0	3	2	4	F
A217	0.478	0.076	0.402	80.7	4	4	33	F
A218	0.322	0.076	0.246	51.1	3	4	66	M
A219	0.444	0.051	0.393	79.0	4	4	4	F
A220	0.361	0.078	0.283	58.2	5	5	63	M
A221	0.315	0.028	0.287	58.9	5	5	50	F
A222	0.337	0.075	0.262	54.2	4	4	53	F
A223	0.186	0.079	0.107	24.8	2	2	23	M
A224	0.327	0.06	0.267	55.1	3	3	42	F
A225	0.327	0.149	0.178	38.2	3	3	42	F
A226	0.345	0.117	0.228	47.7	3	3	68	M
A227	0.558	0.084	0.474	94.4	4	4	30	F
A228	0.417	0.054	0.363	73.3	4	4	21	M
A229	1.07	0.419	0.651	127.9	6	6	38	M
A230	0.467	0.068	0.399	80.1	4	4	35	F
A231	0.217	0.052	0.165	35.8	2	2	45	F
A232	0.455	0.076	0.379	76.3	4	4	42	F
A233	0.489	0.085	0.404	81.1	5	5	2	M

A234	0.547	0.38	0.167	36.2	5	5	37	M
A235	0.489	0.103	0.386	77.7	5	5	59	M
A236	0.523	0.042	0.481	95.7	5	5	49	F
A237	0.364	0.075	0.289	59.3	3	3	49	F
A238	0.187	0.071	0.116	26.5	2	2	48	F
A239	0.336	0.072	0.264	54.5	3	3	54	F
A240	0.468	0.095	0.373	75.2	4	4	51	F
A241	0.578	0.089	0.489	97.2	5	5	54	F
A242	0.736	0.07	0.666	130.8	6	6	50	F
A243	0.34	0.089	0.251	52.1	3	3	60	F
A244	0.285	0.089	0.196	41.7	3	3	34	F
A245	0.578	0.065	0.513	101.7	4	4	53	M
A246	0.306	0.069	0.237	49.4	3	3	53	F
A247	0.444	0.072	0.372	75.0	4	4	38	F
A248	0.616	0.086	0.53	105.0	5	5	16	F
A249	0.191	0.074	0.117	26.7	2	2	65	F
A250	0.629	0.094	0.535	105.9	5	5	43	F
A251	0.478	0.292	0.186	39.8	4	4	33	F
A252	0.348	0.12	0.228	47.7	3	3	51	F
A253	0.848	0.076	0.772	150.8	6	6	55	F
A254	0.95	0.064	0.886	172.5	7	7	28	M
A255	0.451	0.11	0.341	69.1	4	4	14	M
A256	0.552	0.158	0.394	79.2	4	4	68	M
A257	0.61	0.104	0.506	100.4	5	5	26	F
A258	0.315	0.109	0.206	43.6	3	3	60	M
A259	0.369	0.154	0.215	45.3	3	3	60	M
A260	0.337	0.188	0.149	32.8	2	3	41	F
A261	1.111	0.257	0.854	166.4	7	7	59	F
A262	0.837	0.63	0.207	43.7	6	6	50	F
A263	0.315	0.085	0.23	48.1	4	3	70	F
A264	0.511	0.097	0.414	83.0	5	5	70	F
A265	0.272	0.075	0.197	41.8	3	3	50	F
A266	0.673	0.104	0.569	112.4	5	5	25	M
A267	0.324	0.085	0.239	49.8	3	3	31	F
A268	0.556	0.187	0.369	74.5	4	4	55	F
A269	0.751	0.078	0.673	132.1	6	6	44	M
A270	0.545	0.067	0.478	95.1	5	5	30	F
A271	0.558	0.085	0.473	94.2	5	5	18	M
A272	0.292	0.067	0.225	47.2	3	3	1yr n 3m	M
**Plate 5**								
**Sample**	**450nm**	**630nm**	**450-630nm**	**%**	**Colour O1**	**Colour O2**	**Age**	**Sex**
Blank	0	0	0		No	No		
Inactivated	0.112	0.081	0.031		No	No		
Sub negative	0.188	0.102	0.086		No	No		
100%	0.735	0.109	0.626		6	6		
60%	0.374	0.063	0.311		4	4		
30%	0.25	0.068	0.182		3	3		
10%	0.181	0.017	0.164		2	2		
A273	0.904	0.215	0.689	115.0	7	7	79	F
A274	0.698	0.245	0.453	73.4	6	6	63	M
A275	0.249	0.051	0.198	28.3	3	3	37	F
A276	1.193	0.264	0.929	157.4	7	7	3y6m	M
A277	1.368	0.426	0.942	159.7	7	7	52	F
A278	0.628	0.046	0.582	96.1	5	5	47	F
A279	0.354	0.108	0.246	36.8	3	3	60	M
A280	0.599	0.081	0.518	84.8	5	5	67	F
A281	0.554	0.049	0.505	82.5	4	4	67	M
A282	0.712	0.269	0.443	71.6	5	5	57	F
A283	0.387	0.057	0.33	51.6	3	3	52	F
A284	0.489	0.122	0.367	58.2	4	4	54	F
A285	0.266	0.052	0.214	31.1	3	3	56	F
A286	0.851	0.1	0.751	126.0	6	6	58	F
A287	0.454	0.1	0.354	55.9	4	4	47	F
A288	0.673	0.058	0.615	102.0	5	5	11	F
A289	1.228	0.225	1.003	170.5	7	7	29	M
A290	0.645	0.047	0.598	99.0	5	5	60	F
A291	0.447	0.05	0.397	63.5	4	4	61	F

A292	0.872	0.114	**0.758**	127.2	6	6	65	M
A293	0.376	0.018	**0.358**	56.6	4	4	28	F
A294	0.376	0.101	**0.275**	41.9	4	4	86	M
A295	0.567	0.032	**0.535**	87.8	4	4	63	F
A296	0.421	0.033	**0.388**	61.9	4	4	56	F
A297	0.59	0.138	**0.452**	73.2	5	5	33	F
A298	0.909	0.123	**0.786**	132.2	6	6	60	F
A299	0.785	0.083	**0.702**	117.3	6	6	49	F
A300	0.504	0.076	**0.428**	68.9	5	5	40	F
A301	0.302	0.082	**0.22**	32.2	3	3	55	F
A302	0.536	0.029	**0.507**	82.9	4	4	21	F
A303	0.452	0.063	**0.389**	62.1	5	5	56	M
A304	0.354	0.078	**0.276**	42.1	3	3	64	M
A305	0.61	0.059	**0.551**	90.7	4	4	49	F
A306	0.47	0.025	**0.445**	71.9	5	5	38	F
A307	0.368	0.037	**0.331**	51.8	3	3	63	M
A308	0.456	0.031	**0.425**	68.4	5	5	74	M
A309	0.509	0.024	**0.485**	79.0	4	4	62	F
A310	0.453	0.067	**0.386**	61.5	4	4	74	F
A311	0.484	0.081	**0.403**	64.5	4	4	64	M
A312	0.72	0.105	**0.615**	102.0	5	5	65	F
A313	0.582	0.032	**0.55**	90.5	5	5	65	F
A314	0.363	0.022	**0.341**	53.6	3	3	62	F
A315	0.259	0.019	**0.24**	35.7	3	3	61	F
A316	0.715	0.072	**0.643**	106.9	6	6	11	M
A317	0.363	0.055	**0.308**	47.7	3	3	60	F
A318	0.582	0.041	**0.541**	88.9	4	4	65	F
A319	0.259	0.027	**0.232**	34.3	3	3	51	F
A320	0.198	0.051	**0.147**	19.3	2	2	35	M
A321	0.401	0.079	**0.322**	50.2	3	3	13	F
A322	0.421	0.092	**0.329**	51.5	3	3	30	F
A323	0.655	0.054	**0.601**	99.5	5	5	65	F
A324	0.805	0.05	**0.755**	126.7	6	6	35	F
A325	0.371	0.031	**0.34**	53.4	3	3	59	M
A326	0.627	0.107	**0.52**	85.2	5	5	37	M
A327	0.34	0.031	**0.309**	47.9	3	3	61	F
A328	0.812	0.079	**0.733**	122.8	6	6	24	M
A329	0.505	0.2	**0.305**	47.2	5	5	5	F
A330	0.557	0.063	**0.494**	80.6	5	5	31	F
A331	0.716	0.106	**0.61**	101.1	6	6	40	M
A332	0.273	0.049	**0.224**	32.9	3	3	34	F
A333	0.46	0.028	**0.432**	69.6	4	4	66	F
A334	0.587	0.079	**0.508**	83.1	5	5	37	F
A335	0.238	0.085	**0.153**	20.4	3	3	63	F
A336	0.337	0.046	**0.291**	44.7	3	3	32	M
A337	0.246	0.041	**0.205**	29.6	3	3	72	M
A338	0.317	0.046	**0.271**	41.2	3	3	48	M
A339	0.432	0.025	**0.407**	65.2	5	5	60	F
A340	0.286	0.071	**0.215**	31.3	3	3	62	M
A341	0.64	0.054	**0.586**	96.8	5	5	59	F
A342	0.385	0.129	**0.256**	38.6	4	4	44	F
A343	0.643	0.055	**0.588**	97.2	5	5	43	F
A344	0.309	0.012	**0.297**	45.8	4	4	74	M
A345	0.594	0.042	**0.552**	90.8	5	5	57	F
A346	0.546	0.056	**0.49**	79.9	5	5	44	F
A347	0.391	0.072	**0.319**	49.7	4	4	21	F
A348	0.286	0.067	**0.219**	32.0	3	3	58	M
A349	0.404	0.07	**0.334**	52.3	4	4	52	F
A350	0.471	0.081	**0.39**	62.2	4	4	73	M
A351	0.313	0.043	**0.27**	41.0	3	3	51	F
A352	0.313	0.06	**0.253**	38.0	3	3	51	M
A353	0.678	0.038	**0.64**	106.4	5	5	65	M
A354	0.336	0.045	**0.291**	44.7	3	3	35	F
A355	0.566	0.049	**0.517**	84.7	5	5	56	F
A356	0.455	0.068	**0.387**	61.7	5	5	52	F
**Plate 6**								
**Sample**	**450nm**	**630nm**	**450-630nm**	**%**	**Colour O1**	**Colour O2**	**Age**	**Sex**

Blank	**0.02**	**0.02**	**0**		**No**	**No**		
Inactivated	**0.178**	**0.124**	**0.054**		**No**	**No**		
Sub negative	**0.169**	**0.153**	**0.016**		**No**	**No**		
100%	**0.636**	**0.094**	**0.542**		**5**	**5**		
60%	**0.346**	**0.097**	**0.249**		**4**	**4**		
30%	**0.308**	**0.128**	**0.18**		**3**	**3**		
10%	**0.251**	**0.101**	**0.15**		**2**	**2**		
A357	0.667	0.258	**0.409**	76.7	5	5	76	M
A358	0.512	0.319	**0.193**	31.9	3	3	59	F
A359	1.169	0.241	**0.928**	184.2	7	7	50	M
A360	0.529	0.093	**0.436**	82.3	5	5	54	F
A361	0.422	0.13	**0.292**	52.4	5	5	52	M
A362	0.407	0.172	**0.235**	40.6	5	5	52	F
A363	0.357	0.097	**0.26**	45.8	5	5	50	M
A364	0.545	0.136	**0.409**	76.7	3	4	45	M
A365	0.677	0.125	**0.552**	106.3	5	5	70	M
A366	0.34	0.085	**0.255**	44.8	3	4	56	F
A367	0.654	0.135	**0.519**	99.5	5	5	47	M
A368	0.286	0.112	**0.174**	28.0	2	3	33	M
A369	0.495	0.144	**0.351**	64.7	4	4	48	F
A370	0.58	0.135	**0.445**	84.1	5	5	62	M
A371	0.351	0.141	**0.21**	35.4	3	3	52	F
A372	0.451	0.119	**0.332**	60.7	5	4	45	F
A373	0.389	0.214	**0.175**	28.2	4	3	50	F
A374	0.385	0.103	**0.282**	50.4	3	3	11	M
A375	0.237	0.156	**0.081**	8.7	2	2	60	F
A376	0.324	0.069	**0.255**	44.8	3	3	63	F
A377	0.392	0.115	**0.277**	49.3	4	4	54	F
A378	0.474	0.181	**0.293**	52.6	4	4	52	M
A379	0.655	0.163	**0.492**	93.9	5	5	50	F
A380	0.336	0.112	**0.224**	38.3	4	3	4y6m	M
A381	0.483	0.094	**0.389**	72.5	5	5	46	F
A382	0.322	0.109	**0.213**	36.1	3	3	39	F
A383	0.331	0.054	**0.277**	49.3	3	3	12	M
A384	0.501	0.062	**0.439**	82.9	5	5	42	M
A385	0.419	0.108	**0.311**	56.4	4	4	20	M
A386	0.716	0.189	**0.527**	101.1	5	5	3	M
A387	0.781	0.147	**0.634**	123.3	6	6	41	F
A388	1.017	0.307	**0.71**	139.0	7	7	48	F
A389	0.293	0.11	**0.183**	29.9	4	3	47	F
A390	0.325	0.112	**0.213**	36.1	3	3	53	F
A391	0.775	0.264	**0.511**	97.8	5	5	66	M
A392	0.285	0.102	**0.183**	29.9	3	3	55	F
A393	0.472	0.117	**0.355**	65.5	5	5	48	M
A394	0.511	0.123	**0.388**	72.3	5	5	18	F
A395	0.722	0.106	**0.616**	119.5	5	5	42	F
A396	0.991	0.416	**0.575**	111.1	6	6	35	F
A397	0.445	0.07	**0.375**	69.6	4	4	1y4m	M
A398	0.725	0.142	**0.583**	112.7	5	5	21	M
A399	0.246	0.164	**0.082**	8.9	2	2	63	F
A400	0.971	0.074	**0.897**	177.8	6	6	3	M
A401	0.562	0.114	**0.448**	84.7	5	5	39	M
A402	0.336	0.15	**0.186**	30.5	4	4	25	F
A403	0.729	0.187	**0.542**	104.2	5	5	10	F
A404	0.723	0.145	**0.578**	111.7	5	5	16	M
A405	0.427	0.099	**0.328**	59.9	4	4	20	F
A406	0.614	0.229	**0.385**	71.7	5	5	59	F
A407	0.243	0.158	**0.085**	9.6	2	2	45	F
A408	0.409	0.078	**0.331**	60.5	4	4	52	F
A409	0.344	0.115	**0.229**	39.4	5	4	35	M
A410	0.859	0.23	**0.629**	122.2	6	6	49	F
A411	0.748	0.137	**0.611**	118.5	5	5	17	F
A412	0.483	0.1	**0.383**	71.3	5	5	42	F
A413	0.944	0.203	**0.741**	145.4	6	6	26	F
A414	1.252	0.359	**0.893**	176.9	7	7	32	F
A415	0.453	0.08	**0.373**	69.2	5	5	32	F
A416	0.508	0.132	**0.376**	69.8	5	5	67	F
A417	0.933	0.292	**0.641**	124.7	6	6	50	F

A418	0.282	0.145	0.137	20.3	4	3	1	M
A419	0.812	0.2	0.612	118.7	5	5	19	M
A420	0.373	0.149	0.224	38.3	4	4	29	M
A421	0.364	0.114	0.25	43.7	4	4	45	M
A422	0.634	0.135	0.499	95.3	5	5	8	F
A423	0.347	0.102	0.245	42.7	4	4	3	M
A424	0.541	0.098	0.443	83.7	5	5	20	F
A425	0.325	0.096	0.229	39.4	4	4	20	F
A426	0.643	0.144	0.499	95.3	5	5	55	F
A427	0.387	0.119	0.268	47.5	4	4	31	F
A428	0.395	0.141	0.254	44.6	4	4	35	F
A429	0.609	0.197	0.412	77.3	5	5	40	M
A430	0.423	0.107	0.316	57.4	3	3	60	F
A431	0.636	0.245	0.391	72.9	5	5	40	F
A432	0.982	0.76	0.222	37.9	3	3	10	F
A433	0.886	0.155	0.731	143.4	6	6	50	F
A434	0.516	0.156	0.36	66.5	4	4	21	F
A435	0.525	0.111	0.414	77.7	5	5	53	F
A436	0.269	0.079	0.19	31.3	4	3	34	F
A437	0.317	0.121	0.196	32.5	4	3	40	F
A438	0.605	0.161	0.444	83.9	4	3	54	F
A439	0.451	0.114	0.337	61.8	5	5	11	M
A440	0.284	0.102	0.182	29.6	3	3	55	F
**Plate 7**								
**Sample**	**450nm**	**630nm**	**450-630nm**	**%**	**Colour O1**	**Colour O2**	**Age**	**Sex**
Blank	0.026	0.025	0.001		No	No		
Inactivated	0.059	0.05	0.009		No	No		
Sub negative	0.061	0.06	0.001		No	No		
100%	1.052	0.145	0.907		7	7		
60%	0.454	0.021	0.433		5	5		
30%	0.176	0.023	0.153		2	2		
10%	0.07	0.033	0.037		1	1		
A441	0.366	0.119	0.247	36.4	3	3	24	M
A442	0.238	0.04	0.198	31.5	3	3	73	F
A443	0.384	0.05	0.334	45.1	3	3	35	F
A444	0.699	0.268	0.431	54.9	3	3	60	M
A445	0.764	0.051	0.713	83.2	5	5	48	F
A446	1.049	0.409	0.64	75.8	6	6	23	F
A447	0.655	0.118	0.537	65.5	5	5	65	M
A448	0.924	0.171	0.753	87.2	5	5	36	F
A449	0.624	0.364	0.26	37.7	5	5	50	F
A450	0.328	0.132	0.196	31.3	3	3	54	F
A451	0.167	0.103	0.064	18.0	3	3	46	F
A452	0.137	0.01	0.127	24.4	3	3	3 8\12	F
A453	0.398	0.046	0.352	46.9	4	4	45	M
A454	0.678	0.088	0.59	70.8	5	5	18	M
A455	1.091	0.302	0.789	90.8	6	6	65	F
A456	0.551	0.121	0.43	54.8	4	5	61	F
A457	0.446	0.226	0.22	33.7	3	3	12	M
A458	0.439	0.093	0.346	46.3	3	3	47	M
A459	0.574	0.012	0.562	68.0	4	4	66	M
A460	1.117	0.498	0.619	73.7	7	7	8	M
A461	0.157	0.045	0.112	22.8	3	3	63	F
A462	0.457	0.043	0.414	53.2	4	3	54	M
A463	0.672	0.193	0.479	59.7	5	5	62	F
A464	0.389	0.197	0.192	30.9	3	3	64	F
A465	0.473	0.036	0.437	55.5	4	4	37	M
A466	0.609	0.28	0.329	44.6	3	3	14	M
A467	0.199	0.012	0.187	30.4	3	3	56	F
A468	0.453	0.173	0.28	39.7	3	3	19	F
A469	0.431	0.04	0.391	50.9	4	3	46	M
A470	0.276	0.087	0.189	30.6	3	3	40	M
A471	0.205	0.015	0.19	30.7	3	3	55	F
A472	0.259	0.038	0.221	33.8	3	3	23	M
A473	0.511	0.168	0.343	46.0	3	3	49	M
A474	0.287	0.04	0.247	36.4	3	3	52	F
A475	0.172	0.049	0.123	24.0	3	3	66	M

A476	0.65	0.192	**0.458**	57.6	3	3	48	M
A477	0.386	0.079	**0.307**	42.4	4	3	33	F
A478	0.313	0.126	**0.187**	30.4	3	3	38	F
A479	0.175	0.04	**0.135**	25.2	3	3	30	F
A480	0.236	0.05	**0.186**	30.3	3	3	67	F
A481	0.864	0.533	**0.331**	44.8	3	3	57	F
A482	0.209	0.021	**0.188**	30.5	3	3	18	F
A483	0.577	0.314	**0.263**	38.0	3	3	63	F
A484	0.422	0.153	**0.269**	38.6	3	3	78	M
A485	0.254	0.058	**0.196**	31.3	3	3	9	M
A486	0.273	0.044	**0.229**	34.6	3	3	54	F
A487	0.446	0.058	**0.388**	50.6	4	4	65	M
A488	0.494	0.217	**0.277**	39.4	3	3	65	F
A489	0.515	0.133	**0.382**	49.9	4	4	64	F
A490	1.269	0.518	**0.751**	87.0	6	6	42	F
A491	0.815	0.409	**0.406**	52.4	5	5	52	F
A492	0.628	0.139	**0.489**	60.7	5	5	1	F
A493	1.667	0.801	**0.866**	98.5	6	6	50	F
A494	0.375	0.168	**0.207**	32.4	3	3	41	F
A495	0.722	0.151	**0.571**	68.9	5	5	72	F
A496	0.733	0.124	**0.609**	72.7	5	5	53	M
A497	0.541	0.034	**0.507**	62.5	5	5	47	F
A498	0.891	0.106	**0.785**	90.4	6	6	1y5m	F
A499	0.826	0.143	**0.683**	80.2	5	5	54	F
A500	0.457	0.036	**0.421**	53.9	5	4	57	F
A501	0.28	0.049	**0.231**	34.8	3	3	60	F
A502	0.284	0.095	**0.189**	30.6	3	3	54	F
A503	0.28	0.06	**0.22**	33.7	3	3	74	M
A504	0.696	0.393	**0.303**	42.0	4	4	60	F
A505	1.288	0.039	**1.249**	137.0	6	6	55	F
A506	0.827	0.199	**0.628**	74.6	5	5	65	M
A507	0.246	0.025	**0.221**	33.8	3	3	58	F
A508	0.386	0.188	**0.198**	31.5	3	3	9m	M
A509	0.49	0.112	**0.378**	49.5	3	3	16	F
A510	0.342	0.071	**0.271**	38.8	3	3	65	M
A511	0.321	0.102	**0.219**	33.6	3	4	13	M
A512	1.145	0.738	**0.407**	52.5	4	4	51	F
A513	0.936	0.438	**0.498**	61.6	5	4	47	M
A514	0.396	0.131	**0.265**	38.2	3	3	1y9m	M
A515	0.368	0.041	**0.327**	44.4	3	3	52	M
A516	0.439	0.113	**0.326**	44.3	3	3	53	M
A517	0.493	0.15	**0.343**	46.0	3	3	54	F
A518	0.245	0.051	**0.194**	31.1	3	3	34	F
A519	1.01	0.339	**0.671**	79.0	4	5	35	F
A520	0.376	0.062	**0.314**	43.1	3	3	53	F
A521	0.873	0.289	**0.584**	70.2	5	5	41	F
A522	0.463	0.149	**0.314**	43.1	3	3	51	F
A523	0.765	0.303	**0.462**	58.0	5	5	1y8m	F
A524	0.492	0.046	**0.446**	56.4	3	4	46	F
**Plate 8**								
**Sample**	**450nm**	**630nm**	**450-630nm**	**%**	**Colour O1**	**Colour O2**	**Age**	**Sex**
Blank	**0.021**	**0.021**	**0**		**No**	**No**		
Inactivated	**0.173**	**0.115**	**0.058**		**No**	**No**		
Sub negative	**0.288**	**0.167**	**0.121**		**No**	**No**		
100%	**0.986**	**0.149**	**0.837**		**6**	**6**		
60%	**0.519**	**0.127**	**0.392**		**4**	**4**		
30%	**0.246**	**0.08**	**0.166**		**3**	**3**		
10%	**0.205**	**0.053**	**0.152**		**2**	**2**		
A525	0.341	0.09	**0.251**	33.9	4	3	54	F
A526	0.536	0.207	**0.329**	43.1	4	4	58	M
A527	0.489	0.142	**0.347**	45.3	4	3	63	M
A528	0.333	0.071	**0.262**	35.2	4	4	9days	M
A529	0.407	0.102	**0.305**	40.3	4	4	64	F
A530	0.385	0.073	**0.312**	41.1	4	4	58	M
A531	0.403	0.065	**0.338**	44.2	4	4	60	F
A532	0.353	0.062	**0.291**	38.6	3	3	43	F
A533	0.701	0.103	**0.598**	75.1	5	5	15	M

A534	0.539	0.145	**0.394**	50.9	4	4	2m 21d	F
A535	0.286	0.054	**0.232**	31.6	3	3	22	F
A536	0.52	0.068	**0.452**	57.7	4	4	29	F
A537	0.588	0.24	**0.348**	45.4	4	4	54	F
A538	0.528	0.084	**0.444**	56.8	5	5	64	F
A539	0.547	0.064	**0.483**	61.4	5	5	60	F
A540	0.667	0.138	**0.529**	66.9	5	5	49	F
A541	0.71	0.352	**0.358**	46.6	3	3	42	F
A542	0.402	0.07	**0.332**	43.5	4	4	34	F
A543	0.233	0.067	**0.166**	23.8	3	3	50	F
A544	0.271	0.047	**0.224**	30.7	3	3	51	F
A545	0.46	0.1	**0.36**	46.8	4	4	66	M
A546	0.549	0.109	**0.44**	56.3	5	5	32	F
A547	0.396	0.09	**0.306**	40.4	4	4	25	F
A548	0.437	0.191	**0.246**	33.3	3	3	36	F
A549	0.532	0.227	**0.305**	40.3	4	4	61	M
A550	0.807	0.437	**0.37**	48.0	4	4	51	M
A551	0.406	0.169	**0.237**	32.2	3	3	28	F
A552	0.457	0.172	**0.285**	37.9	3	3	84	M
A553	0.345	0.06	**0.285**	37.9	3	3	52	M
A554	0.334	0.092	**0.242**	32.8	3	3	72	M
A555	0.343	0.114	**0.229**	31.3	3	3	74	F
A556	0.361	0.049	**0.312**	41.1	3	3	18	M
A557	0.462	0.095	**0.367**	47.7	4	4	47	F
A558	0.236	0.011	**0.225**	30.8	3	3	36	F
A559	0.422	0.116	**0.306**	40.4	3	3	20	F
A560	0.379	0.117	**0.262**	35.2	3	3	10	M
A561	1.138	0.863	**0.275**	36.7	3	3	72	F
A562	0.514	0.128	**0.386**	49.9	4	4	8	F
A563	0.766	0.474	**0.292**	38.8	3	3	4y6m	F
A564	0.654	0.126	**0.528**	66.8	5	5	56	F
A565	0.72	0.394	**0.326**	42.8	4	4	35	F
A566	0.313	0.069	**0.244**	33.1	3	3	48	F
A567	0.563	0.096	**0.467**	59.5	4	4	65	M
A568	1.203	0.607	**0.596**	74.8	5	5	49	F
A569	0.377	0.098	**0.279**	37.2	4	4	60	F
A570	0.633	0.148	**0.485**	61.7	4	4	1y2m	M
A571	1.158	0.918	**0.24**	32.6	3	3	2y9m	M
A572	0.953	0.244	**0.709**	88.2	6	6	6	F
A573	0.764	0.194	**0.57**	71.7	5	5	49	F
A574	0.799	0.115	**0.684**	85.3	5	5	56	F
A575	0.251	0.021	**0.23**	31.4	3	3	35	F
A576	0.282	0.059	**0.223**	30.6	3	3	61	F
A577	0.511	0.128	**0.383**	49.6	4	4	19	F
A578	0.599	0.369	**0.23**	31.4	3	3	32	F
A579	0.223	0.109	**0.114**	17.6	3	3	41	F
A580	0.285	0.059	**0.226**	30.9	3	3	59	M
A581	0.356	0.131	**0.225**	30.8	3	3	19	F
A582	0.417	0.163	**0.254**	34.3	3	3	26	F
A583	0.468	0.135	**0.333**	43.6	3	3	39	M
A584	0.954	0.402	**0.552**	69.6	4	4	62	M
A585	0.914	0.098	**0.816**	100.9	5	5	31	F
A586	1.243	0.572	**0.671**	83.7	5	5	48	F
A587	1.184	0.681	**0.503**	63.8	5	5	9mon	F
A588	0.506	0.08	**0.426**	54.7	5	5	58	F
A589	0.38	0.14	**0.24**	32.6	3	3	6	F
A590	1.183	0.527	**0.656**	81.9	5	5	51	F
A591	0.688	0.31	**0.378**	49.0	3	3	43	F
A592	1.197	0.519	**0.678**	84.6	5	5	52	F
A593	0.305	0.081	**0.224**	30.7	3	3	35	F
A594	0.535	0.221	**0.314**	41.4	3	3	63	F
A595	0.828	0.418	**0.41**	52.8	3	3	48	F
A596	1.312	0.424	**0.888**	109.5	5	5	55	F
A597	0.427	0.155	**0.272**	36.4	3	3	64	M
A598	1.948	0.944	**1.004**	123.2	6	6	8	M
A599	0.503	0.107	**0.396**	51.1	3	3	8	F
A600	0.496	0.141	**0.355**	46.2	4	4	71	F
A601	0.931	0.629	**0.302**	39.9	3	3	43	F

A602	0.356	0.133	**0.223**	30.6	3	3	28	F
A603	0.864	0.274	**0.59**	74.1	5	5	79	F
A604	0.317	0.082	**0.235**	32.0	3	3	58	F
A605	0.491	0.087	**0.404**	52.0	3	3	1	M
A606	0.343	0.066	**0.277**	37.0	3	3	30	M
A607	0.366	0.082	**0.284**	37.8	3	3	61	M
A608	1.65	0.937	**0.713**	88.7	6	6	36	M
**Plate 9**								
**Sample**	**450nm**	**630nm**	**450-630nm**	**%**	**Colour O1**	**Colour O2**	**Age**	**Sex**
Blank	**0.02**	**0.02**	**0**		**No**	**No**		
Inactivated	**0.138**	**0.11**	**0.028**		**No**	**No**		
Sub negative	**0.2**	**0.022**	**0.178**		**No**	**No**		
100%	**0.729**	**0.053**	**0.676**		**5**	**5**		
60%	**0.416**	**0.052**	**0.364**		**4**	**4**		
30%	**0.246**	**0.044**	**0.202**		**3**	**3**		
10%	**0.157**	**0.048**	**0.109**		**2**	**2**		
A609	0.669	0.329	**0.34**	50.4	3	4	52	F
A610	0.485	0.116	**0.369**	54.9	5	5	65	F
A611	1.061	0.274	**0.787**	120.2	6	6	51	F
A612	0.499	0.125	**0.374**	55.7	5	5	65	F
A613	1.064	0.415	**0.649**	98.6	5	5	58	F
A614	0.745	0.158	**0.587**	88.9	5	5	4 7 / 12	F
A615	0.505	0.206	**0.299**	43.9	3	3	44	F
A616	0.261	0.044	**0.217**	31.1	3	3	58	F
A617	1.189	0.611	**0.578**	87.5	5	5	55	F
A618	0.227	0.073	**0.154**	21.3	3	3	49	M
A619	0.21	0.055	**0.155**	21.4	3	3	60	M
A620	0.536	0.064	**0.472**	71.0	3	4	20	F
A621	0.28	0.062	**0.218**	31.3	3	3	32	F
A622	0.849	0.172	**0.677**	103.0	5	5	26	F
A623	0.51	0.079	**0.431**	64.6	4	3	64	F
A624	0.455	0.118	**0.337**	49.9	3	3	26	M
A625	0.306	0.064	**0.242**	35.0	3	3	1 6 / 12	M
A626	0.328	0.086	**0.242**	35.0	3	3	68	M
A627	0.336	0.068	**0.268**	39.1	3	3	24	F
A628	0.359	0.069	**0.29**	42.5	3	3	34	M
A629	0.2	0.085	**0.115**	15.2	3	3	59	M
A630	0.421	0.124	**0.297**	43.6	3	3	41	F
A631	0.423	0.09	**0.333**	49.3	3	3	57	F
A632	0.436	0.148	**0.288**	42.2	3	3	44	F
A633	0.528	0.086	**0.442**	66.3	3	4	29	M
A634	0.347	0.094	**0.253**	36.8	3	3	56	F
A635	0.557	0.085	**0.472**	71.0	5	5	50	M
A636	0.331	0.109	**0.222**	31.9	3	3	62	F
A637	0.298	0.086	**0.212**	30.4	3	3	10	M
A638	0.638	0.121	**0.517**	78.0	5	5	68	F
A639	0.295	0.081	**0.214**	30.7	3	3	53	F
A640	0.398	0.141	**0.257**	37.4	3	3	43	F
A641	0.331	0.106	**0.225**	32.4	4	4	53	F
A642	0.6	0.178	**0.422**	63.2	4	4	37	M
A643	1.123	0.065	**1.058**	162.5	7	7	33	F
A644	0.36	0.072	**0.288**	42.2	3	4	72	F
A645	0.562	0.094	**0.468**	70.4	4	4	64	M
A646	0.654	0.139	**0.515**	77.7	5	5	68	M
A647	0.403	0.118	**0.285**	41.8	4	4	54	M
A648	0.645	0.163	**0.482**	72.5	4	4	62	M
A649	0.9	0.123	**0.777**	118.6	5	5	38	M
A650	0.577	0.097	**0.48**	72.2	4	4	53	F
A651	0.776	0.078	**0.698**	106.3	5	5	65	F
A652	0.538	0.128	**0.41**	61.3	4	4	43	F
A653	0.363	0.107	**0.256**	37.2	3	3	65	M
A654	0.329	0.102	**0.227**	32.7	3	3	70	M
A655	0.44	0.106	**0.334**	49.4	3	3	14	M
A656	0.419	0.137	**0.282**	41.3	3	3	52	M
A657	0.456	0.171	**0.285**	41.8	3	3	56	F
A658	0.428	0.211	**0.217**	31.1	4	4	7	M
A659	1.276	0.09	**1.186**	182.5	7	7	63	F

A660	0.539	0.107	**0.432**	64.7	4	4	43	M
A661	0.831	0.106	**0.725**	110.5	5	5	40	M
A662	0.359	0.106	**0.253**	36.8	3	3	35	F
A663	0.489	0.118	**0.371**	55.2	3	3	61	M
A664	0.255	0.039	**0.216**	31.0	4	4	66	F
A665	0.662	0.111	**0.551**	83.3	4	4	73	M
A666	0.417	0.201	**0.216**	31.0	3	4	1 3 /12	M
A667	0.52	0.136	**0.384**	57.2	4	4	69	F
A668	0.713	0.151	**0.562**	85.0	4	4	52	F
A669	0.893	0.14	**0.753**	114.9	5	5	21	M
A670	0.352	0.098	**0.254**	36.9	3	3	61	F
A671	0.694	0.127	**0.567**	85.8	5	5	50	F
A672	0.321	0.105	**0.216**	31.0	4	4	14	F
A673	0.83	0.113	**0.717**	109.3	5	5	72	F
A674	0.628	0.132	**0.496**	74.7	3	4	44	F
A675	0.46	0.112	**0.348**	51.6	4	4	68	F
A676	0.629	0.123	**0.506**	76.3	5	5	58	F
A677	0.322	0.109	**0.213**	30.5	4	4	64	F
A678	0.727	0.187	**0.54**	81.6	4	4	53	M
A679	0.873	0.131	**0.742**	113.2	5	5	61	M
A680	0.297	0.083	**0.214**	30.7	3	3	81	F
A681	0.359	0.126	**0.233**	33.6	3	3	4	M
A682	0.25	0.016	**0.234**	33.8	4	4	70	F
A683	0.835	0.103	**0.732**	111.6	4	4	72	F
A684	0.344	0.126	**0.218**	31.3	3	3	1 y 5mon	M
A685	0.313	0.094	**0.219**	31.4	4	3	48	F
A686	0.467	0.176	**0.291**	42.7	3	3	67	F
A687	0.339	0.119	**0.22**	31.6	3	3	75	M
A688	0.402	0.16	**0.242**	35.0	3	3	21	F
A689	0.417	0.132	**0.285**	41.8	3	3	10mon	M
A690	0.491	0.153	**0.338**	50.0	3	4	51	F
A691	0.307	0.093	**0.214**	30.7	3	3	61	F
A692	0.427	0.176	**0.251**	36.4	3	3	55	F

**Plate 10**								
**Sample**	**450nm**	**630nm**	**450-630nm**	**%**	**Colour O1**	**Colour O2**	**Age**	**Sex**
Blank	**0.02**	**0.02**	**0**		**No**	**No**		
Inactivated	**0.096**	**0.063**	**0.033**		**No**	**No**		
Sub negative	**0.11**	**0.09**	**0.02**		**No**	**No**		
100%	**0.437**	**0.054**	**0.383**		**4**	**4**		
60%	**0.385**	**0.109**	**0.276**		**3**	**3**		
30%	**0.128**	**0.059**	**0.069**		**2**	**2**		
10%	**0.107**	**0.067**	**0.04**		**1**	**1**		
A693	0.344	0.087	**0.257**	65.0	3	3	34	F
A694	0.757	0.085	**0.672**	161.1	5	5	37	M
A695	0.756	0.35	**0.406**	99.5	5	5	48	M
A696	0.322	0.124	**0.198**	51.4	3	3	49	M
A697	0.174	0.05	**0.124**	34.3	3	3	31	F
A698	0.462	0.05	**0.412**	100.9	4	4	26	M
A699	0.184	0.077	**0.107**	30.3	3	3	63	F
A700	0.691	0.26	**0.431**	105.3	4	4	81	F
A701	0.387	0.177	**0.21**	54.2	3	3	52	F
A702	0.461	0.098	**0.363**	89.6	4	4	52	F
A703	0.823	0.053	**0.77**	183.8	6	6	3	M
A704	0.461	0.036	**0.425**	103.9	5	5	45	F
A705	0.193	0.0386	**0.1544**	41.3	3	3	46	F
A706	0.49	0.054	**0.436**	106.5	5	5	37	M
A707	0.341	0.058	**0.283**	71.1	3	3	48	F
A708	0.317	0.048	**0.269**	67.8	3	3	7	M
A709	0.219	0.054	**0.165**	43.7	3	3	8	F
A710	0.451	0.06	**0.391**	96.1	5	5	20	M
A711	0.442	0.044	**0.398**	97.7	5	5	20	M
A712	0.409	0.057	**0.352**	87.0	4	4	21	M
A713	0.244	0.059	**0.185**	48.4	4	4	2y11m	F
A714	0.4	0.071	**0.329**	81.7	3	4	21	M
A715	0.353	0.047	**0.306**	76.4	4	4	37	M
A716	0.333	0.038	**0.295**	73.8	3	3	66	M
A717	0.385	0.043	**0.342**	84.7	4	4	21	M

A718	0.379	0.047	**0.332**	82.4	4	4	45	M
A719	0.445	0.074	**0.371**	91.4	4	4	1	M
A720	0.286	0.032	**0.254**	64.4	3	3	1y6m	M
A721	0.31	0.049	**0.261**	66.0	4	4	40	F
A722	0.236	0.054	**0.182**	47.7	3	3	1	M
A723	0.411	0.083	**0.328**	81.5	4	4	51	M
A724	0.253	0.044	**0.209**	53.9	3	3	58	F
A725	0.326	0.047	**0.279**	70.1	3	3	37	F
A726	0.172	0.062	**0.11**	31.0	3	3	26	F
A727	0.486	0.046	**0.44**	107.4	4	4	54	F
A728	0.226	0.018	**0.208**	53.7	3	3	18	M
A729	0.192	0.052	**0.14**	38.0	3	3	22	F
A730	0.308	0.064	**0.244**	62.0	3	3	49	F
A731	0.297	0.064	**0.233**	59.5	3	3	24	F
A732	0.369	0.041	**0.328**	81.5	4	4	50	F
A733	0.337	0.032	**0.305**	76.2	4	4	2	M
A734	0.289	0.053	**0.236**	60.2	3	3	26	F
A735	0.26	0.084	**0.176**	46.3	3	3	52	F
A736	0.402	0.089	**0.313**	78.0	4	4	2	F
A737	0.266	0.046	**0.22**	56.5	3	3	64	F
A738	0.237	0.049	**0.188**	49.1	3	3	60	F
A739	0.366	0.062	**0.304**	75.9	4	4	52	F
A740	0.353	0.045	**0.308**	76.9	4	4	38	F
A741	0.31	0.065	**0.245**	62.3	3	3	21	F
A742	0.339	0.064	**0.275**	69.2	3	3	60	F
A743	0.249	0.046	**0.203**	52.5	3	3	34	F
A744	0.339	0.032	**0.307**	76.6	4	4	44	F
A745	0.144	0.035	**0.109**	30.8	3	3	70	F
A746	0.26	0.055	**0.205**	53.0	3	3	3y4m	M
A747	0.341	0.044	**0.297**	74.3	3	3	73	F
A748	0.477	0.043	**0.434**	106.0	4	4	31	F
A749	0.294	0.041	**0.253**	64.1	3	3	52	F
A750	0.27	0.16	**0.11**	31.0	3	3	57	M
A751	0.534	0.212	**0.322**	80.1	4	4	57	F
A752	0.689	0.031	**0.658**	157.9	5	5	1y3m	M
A753	0.096	0.078	**0.018**	9.7	2	2	5	F
A754	0.201	0.058	**0.143**	38.7	3	3	68	F
A755	0.218	0.068	**0.15**	40.3	3	3	30	F
A756	0.298	0.056	**0.242**	61.6	3	3	44	F
A757	0.308	0.087	**0.221**	56.7	3	4	11	F
A758	0.297	0.047	**0.25**	63.4	3	3	2y5m	F
A759	0.236	0.059	**0.177**	46.5	3	3	11	F
A760	0.121	0.043	**0.078**	23.6	3	3	62	F
A761	0.175	0.057	**0.118**	32.9	3	3	62	F
A762	0.162	0.053	**0.109**	30.8	3	3	49	F
A763	0.236	0.095	**0.141**	38.2	3	3	70	M
A764	0.347	0.24	**0.107**	30.3	4	4	33	F
A765	0.531	0.076	**0.455**	110.9	5	5	75	M
A766	0.189	0.081	**0.108**	30.6	3	3	24	F
A767	0.336	0.1	**0.236**	60.2	3	3	46	M
A768	0.205	0.037	**0.168**	44.4	3	3	49	F
A769	0.163	0.053	**0.11**	31.0	3	3	49	F
A770	0.152	0.037	**0.115**	32.2	3	3	19	F
A771	0.236	0.053	**0.183**	47.9	3	3	22	F
A772	0.364	0.109	**0.255**	64.6	3	3	39	F
A773	0.292	0.069	**0.223**	57.2	4	4	21	F
A774	0.355	0.148	**0.207**	53.5	4	4	55	M
A775	0.687	0.068	**0.619**	148.9	5	5	40	M
A776	0.297	0.068	**0.229**	58.6	3	3	56	F

**Plate 11**								
**Sample**	**450nm**	**630nm**	**450-630nm**	**%**	**Colour O1**	**Colour O2**	**Age**	**Sex**
Blank	**0.01**	**0.01**	**0**		**No**	**No**		
Inactivated	**0.132**	**0.098**	**0.034**		**No**	**No**		
Sub negative	**0.112**	**0.077**	**0.035**		**No**	**No**		
100%	**0.478**	**0.098**	**0.38**		**5**	**5**		
60%	**0.361**	**0.115**	**0.246**		**4**	**4**		
30%	**0.211**	**0.087**	**0.124**		**3**	**3**		

10%	**0.13**	**0.074**	**0.056**		**2**	**2**		
A777	0.23	0.098	**0.132**	30.9	3	3	1	F
A778	0.342	0.065	**0.277**	70.7	4	4	70	M
A779	0.279	0.12	**0.159**	38.3	3	3	23	F
A780	0.252	0.121	**0.131**	30.7	3	3	53	F
A781	0.172	0.044	**0.128**	29.8	3	3	49	M
A782	0.201	0.075	**0.126**	29.3	3	3	6	M
A783	0.251	0.12	**0.131**	30.7	3	3	54	F
A784	0.44	0.108	**0.332**	85.8	4	4	49	M
A785	0.384	0.092	**0.292**	74.8	4	4	25	F
A786	0.201	0.086	**0.115**	26.3	3	3	54	F
A787	0.17	0.058	**0.112**	25.4	3	3	60	F
A788	0.286	0.095	**0.191**	47.1	3	3	33	F
A789	0.196	0.079	**0.117**	26.8	3	3	2	F
A790	0.79	0.102	**0.688**	183.5	5	5	55	F
A791	0.229	0.095	**0.134**	31.5	4	4	7	M
A792	0.439	0.065	**0.374**	97.3	3	4	62	F
A793	0.189	0.075	**0.114**	26.0	3	3	45	F
A794	0.221	0.088	**0.133**	31.2	3	3	67	F
A795	0.249	0.11	**0.139**	32.9	3	3	74	F
A796	0.301	0.109	**0.192**	47.4	3	4	1y5m	F
A797	0.173	0.048	**0.125**	29.0	3	3	28	F
A798	0.216	0.06	**0.156**	37.5	3	3	57	F
A799	0.42	0.23	**0.19**	46.8	3	3	50	F
A800	0.207	0.108	**0.099**	21.9	3	3	74	M
A801	0.117	0.088	**0.029**	2.7	2	2	24	F
A802	0.278	0.143	**0.135**	31.8	3	3	65	F
A803	0.403	0.148	**0.255**	64.7	4	4	60	M
A804	0.421	0.137	**0.284**	72.6	4	4	34	F
A805	0.151	0.067	**0.084**	17.8	3	3	22	F
A806	0.207	0.104	**0.103**	23.0	4	4	23	F
A807	0.207	0.11	**0.097**	21.3	3	3	45	M
A808	0.142	0.076	**0.066**	12.8	3	3	39	F
A809	0.206	0.079	**0.127**	29.6	3	3	52	F
A810	0.227	0.089	**0.138**	32.6	3	3	52	F
A811	0.477	0.056	**0.421**	110.2	5	5	40	F
A812	0.189	0.083	**0.106**	23.8	3	3	60	F
A813	0.266	0.071	**0.195**	48.2	4	4	56	M
A814	0.215	0.084	**0.131**	30.7	3	3	69	M
A815	0.206	0.081	**0.125**	29.0	3	3	70	F
A816	0.195	0.124	**0.071**	14.2	3	3	0.9	M
A817	0.179	0.057	**0.122**	28.2	3	3	19	M
A818	0.187	0.123	**0.064**	12.3	3	3	9	F
A819	0.341	0.102	**0.239**	60.3	4	4	47	M
A820	0.233	0.098	**0.135**	31.8	3	3	32	F
A821	0.173	0.059	**0.114**	26.0	4	4	9	F
A822	0.199	0.074	**0.125**	29.0	3	3	85	M
A823	0.282	0.099	**0.183**	44.9	3	3	66	M
A824	0.258	0.09	**0.168**	40.8	3	3	40	M
A825	0.25	0.067	**0.183**	44.9	3	3	11	M
A826	0.327	0.155	**0.172**	41.9	3	3	55	F
A827	0.104	0.064	**0.04**	5.7	2	2	36	M
A828	0.256	0.112	**0.144**	34.2	3	3	72	M
A829	0.223	0.072	**0.151**	36.1	4	4	34	F
A830	0.195	0.093	**0.102**	22.7	3	3	55	F
A831	0.22	0.094	**0.126**	29.3	3	3	47	F
A832	0.253	0.118	**0.135**	31.8	3	3	20	F
A833	0.248	0.082	**0.166**	40.3	3	3	18	F
A834	0.247	0.093	**0.154**	37.0	3	3	22	M
A835	0.358	0.076	**0.282**	72.1	4	4	47	F
A836	0.337	0.09	**0.247**	62.5	4	4	20	F
A837	0.227	0.069	**0.158**	38.1	4	4	64	F
A838	0.201	0.095	**0.106**	23.8	3	3	56	F
A839	0.492	0.287	**0.205**	51.0	4	4	30	F
A840	0.364	0.107	**0.257**	65.2	3	4	62	F
A841	0.205	0.124	**0.081**	16.9	3	3	52	F
A842	0.429	0.146	**0.283**	72.4	4	4	2y6m	F
A843	0.643	0.146	**0.497**	131.1	5	5	63	M

A844	0.298	0.08	0.218	54.5	3	4	29	F
A845	0.168	0.063	0.105	23.5	3	3	56	F
A846	0.143	0.019	0.124	28.7	3	3	2	F
A847	0.261	0.095	0.166	40.3	4	4	46	F
A848	0.349	0.105	0.244	61.7	4	4	5	M
A849	0.192	0.061	0.131	30.7	3	3	52	F
A850	0.227	0.09	0.137	32.3	3	3	48	F
A851	0.185	0.073	0.112	25.4	3	3	63	F
A852	0.165	0.09	0.075	15.3	3	3	65	F
A853	0.215	0.09	0.125	29.0	3	3	38	F
A854	0.246	0.104	0.142	33.7	3	3	57	F
A855	0.369	0.164	0.205	51.0	3	4	20	F
A856	0.528	0.122	0.406	106.1	4	4	60	F
A857	0.333	0.102	0.231	58.1	3	4	52	M
A858	0.493	0.255	0.238	60.0	3	4	19	F
A859	0.196	0.085	0.111	25.2	3	3	60	F
A860	0.352	0.19	0.162	39.2	3	3	57	F
**Plate 12**								
**Sample**	**450nm**	**630nm**	**450-630nm**	**%**	**Colour O1**	**Colour O2**	**Age**	**Sex**
Blank	0.015	0.015	0		No	No		
Inactivated	0.12	0.088	0.032		No	No		
Sub negative	0.154	0.119	0.035		No	No		
100%	0.609	0.013	0.596		5	5		
60%	0.261	0.013	0.248		4	4		
30%	0.179	0.075	0.104		3	3		
10%	0.14	0.051	0.089		2	2		
A861	0.339	0.08	0.259	50.0	4	4	40	M
A862	0.436	0.119	0.317	59.2	4	4	34	F
A863	0.197	0.046	0.151	32.7	3	3	60	F
A864	0.14	0.099	0.041	15.1	3	3	55	F
A865	0.127	0.045	0.082	21.7	4	4	57	F
A866	0.188	0.045	0.143	31.4	3	3	36	F
A867	0.332	0.195	0.137	30.5	3	3	62	F
A868	0.37	0.173	0.197	40.0	3	4	53	F
A869	0.234	0.078	0.156	33.5	3	3	33	F
A870	0.196	0.05	0.146	31.9	3	3	63	M
A871	0.185	0.04	0.145	31.7	3	3	58	F
A872	0.284	0.088	0.196	39.9	4	4	20	M
A873	0.187	0.048	0.139	30.8	4	4	53	F
A874	0.236	0.07	0.166	35.1	4	4	70	M
A875	0.184	0.041	0.143	31.4	4	4	79	F
A876	0.207	0.063	0.144	31.6	3	3	43	F
A877	0.205	0.067	0.138	30.6	3	3	76	M
A878	0.324	0.051	0.273	52.2	4	4	53	M
A879	0.133	0.058	0.075	20.5	3	3	66	F
A880	0.199	0.056	0.143	31.4	4	4	44	M
A881	0.109	0.107	0.002	8.9	2	2	73	F
A882	0.256	0.085	0.171	35.9	4	4	53	F
A883	0.308	0.102	0.206	41.5	4	4	52	F
A884	0.174	0.095	0.079	21.2	3	3	13	F
A885	0.281	0.069	0.212	42.4	4	4	36	F
A886	0.118	0.11	0.008	9.8	2	2	50	F
A887	0.269	0.106	0.163	34.6	3	3	6y6m	F
A888	0.154	0.104	0.05	16.5	3	3	49	F
A889	0.073	0.069	0.004	9.2	1	1	50	F
A890	0.29	0.15	0.14	30.9	4	4	61	F
A891	0.276	0.065	0.211	42.3	4	4	28	F
A892	0.277	0.075	0.202	40.8	4	4	60	M
A893	0.191	0.051	0.14	30.9	3	3	60	F
A894	0.185	0.048	0.137	30.5	3	3	62	F
A895	0.263	0.071	0.192	39.2	4	4	24	F
A896	0.536	0.057	0.479	85.1	4	4	23	F
A897	0.408	0.073	0.335	62.1	4	4	45	F
A898	0.103	0.1	0.003	9.0	2	2	15	M
A899	0.404	0.201	0.203	41.0	4	4	31	F
A900	0.17	0.143	0.027	12.9	3	3	56	F
A901	0.325	0.125	0.2	40.5	4	4	57	F

A902	0.214	0.071	**0.143**	31.4	3	3	38	F
A903	0.165	0.06	**0.105**	25.3	3	3	18	M
A904	0.545	0.125	**0.42**	75.7	4	4	36	F
A905	0.502	0.36	**0.142**	31.3	4	4	31	F
A906	0.387	0.2	**0.187**	38.4	4	4	34	F
A907	0.189	0.041	**0.148**	32.2	4	4	50	M
A908	0.155	0.098	**0.057**	17.7	4	4	55	F
A909	0.322	0.086	**0.236**	46.3	4	4	52	F
A910	0.31	0.077	**0.233**	45.8	4	4	57	F
A911	0.27	0.095	**0.175**	36.5	4	4	65	F
A912	0.268	0.081	**0.187**	38.4	4	4	10	M
A913	0.122	0.057	**0.065**	18.9	4	4	1 4\12	M
A914	0.33	0.103	**0.227**	44.8	4	4	35	M
A915	0.533	0.134	**0.399**	72.3	4	4	34	F
A916	0.152	0.084	**0.068**	19.4	3	3	74	F
A917	0.23	0.089	**0.141**	31.1	3	3	62	F
A918	0.303	0.125	**0.178**	37.0	3	3	50	F
A919	0.207	0.066	**0.141**	31.1	3	3	42	F
A920	0.19	0.047	**0.143**	31.4	3	3	84	M
A921	0.25	0.07	**0.18**	37.3	4	4	48	F
A922	0.277	0.11	**0.167**	35.3	4	4	47	M
A923	0.268	0.093	**0.175**	36.5	4	4	52	F
A924	0.308	0.119	**0.189**	38.8	4	4	41	F
A925	0.388	0.091	**0.297**	56.0	4	4	60	F
A926	0.245	0.1	**0.145**	31.7	3	3	3 10\12	F
A927	0.152	0.089	**0.063**	18.6	3	3	23	F
A928	0.185	0.046	**0.139**	30.8	3	3	50	F
A929	0.186	0.048	**0.138**	30.6	4	4	6	M
A930	0.196	0.057	**0.139**	30.8	3	3	36	F
A931	0.314	0.115	**0.199**	40.4	4	4	1y3m	M
A932	0.514	0.128	**0.386**	70.3	4	4	48	M
A933	0.225	0.084	**0.141**	31.1	3	3	34	M
A934	0.272	0.122	**0.15**	32.5	3	3	59	F
A935	0.22	0.081	**0.139**	30.8	3	3	17	F
A936	0.172	0.114	**0.058**	17.8	3	3	54	M
A937	0.14	0.102	**0.038**	14.6	3	3	56	F
A938	0.167	0.135	**0.032**	13.7	3	3	44	F
A939	0.171	0.106	**0.065**	18.9	4	4	53	F
A940	0.145	0.123	**0.022**	12.1	3	3	50	F
A941	0.294	0.108	**0.186**	38.3	3	4	23	F
A942	0.39	0.146	**0.244**	47.6	4	4	54	M
A943	0.13	0.096	**0.034**	14.0	3	3	15	F
A944	0.118	0.11	**0.008**	9.8	2	2	50	F

**Plate 13**								
**Sample**	**450nm**	**630nm**	**450-630nm**	**%**	**Colour O1**	**Colour O2**	**Age**	**Sex**
Blank	**0.029**	**0.02**	**0.009**		**No**	**No**		
Inactivated	**0.092**	**0.052**	**0.04**		**No**	**No**		
Sub negative	**0.162**	**0.125**	**0.037**		**No**	**No**		
100%	**0.656**	**0.081**	**0.575**		**5**	**5**		
60%	**0.333**	**0.054**	**0.279**		**4**	**4**		
30%	**0.142**	**0.034**	**0.108**		**3**	**3**		
10%	**0.108**	**0.045**	**0.063**		**2**	**2**		
A945	0.341	0.048	**0.293**	56.1	4	4	10	F
A946	0.647	0.351	**0.296**	56.6	4	4	60	M
A947	0.539	0.04	**0.499**	90.4	5	5	20	M
A948	0.18	0.039	**0.141**	30.8	3	4	37	F
A949	0.138	0.07	**0.068**	18.7	3	4	29	F
A950	0.285	0.061	**0.224**	44.6	3	3	25	M
A951	0.195	0.036	**0.159**	33.8	4	4	30	F
A952	0.39	0.128	**0.262**	51.0	4	4	36	F
A953	0.512	0.095	**0.417**	76.7	5	5	42	F
A954	0.61	0.142	**0.468**	85.2	5	5	40	F
A955	0.422	0.036	**0.386**	71.6	4	4	59	F
A956	0.335	0.026	**0.309**	58.8	4	4	1y4m	M
A957	0.189	0.044	**0.145**	31.5	4	4	51	F
A958	0.153	0.014	**0.139**	30.5	3	3	62	F
A959	0.347	0.195	**0.152**	32.7	3	3	47	M

A960	0.211	0.032	**0.179**	37.1	3	3	51	F
A961	0.303	0.022	**0.281**	54.1	3	3	13	M
A962	0.603	0.199	**0.404**	74.6	4	4	13	F
A963	0.492	0.041	**0.451**	82.4	4	4	62	M
A964	0.211	0.073	**0.138**	30.3	3	4	60	F
A965	0.139	0.061	**0.078**	20.3	3	4	62	M
A966	0.1	0.095	**0.005**	8.2	1	1	62	F
A967	0.54	0.262	**0.278**	53.6	4	4	64	F
A968	0.334	0.06	**0.274**	53.0	4	4	30	F
A969	0.131	0.062	**0.069**	18.8	3	3	50	F
A970	0.184	0.045	**0.139**	30.5	3	3	60	F
A971	0.252	0.095	**0.157**	33.5	3	4	50	F
A972	0.101	0.093	**0.008**	8.7	1	1	63	F
A973	0.119	0.023	**0.096**	23.3	3	4	23	F
A974	0.374	0.107	**0.267**	51.8	3	4	73	F
A975	0.184	0.033	**0.151**	32.5	3	3	52	F
A976	0.234	0.049	**0.185**	38.1	3	3	53	F
A977	0.409	0.27	**0.139**	30.5	3	4	62	F
A978	0.164	0.023	**0.141**	30.8	3	3	31	F
A979	0.321	0.172	**0.149**	32.2	3	4	65	F
A980	0.243	0.095	**0.148**	32.0	3	3	38	M
A981	0.204	0.056	**0.148**	32.0	4	4	34	F
A982	0.074	0.069	**0.005**	8.2	2	2	70	F
A983	0.175	0.033	**0.142**	31.0	3	3	23	F
A984	0.213	0.073	**0.14**	30.7	3	3	60	F
A985	0.519	0.167	**0.352**	65.9	3	4	45	F
A986	0.754	0.287	**0.467**	85.1	5	5	66	F
A987	0.077	0.071	**0.006**	8.4	2	2	61	F
A988	0.26	0.076	**0.184**	38.0		3	68	F
A989	0.257	0.11	**0.147**	31.8	4	4	43	F
A990	0.38	0.069	**0.311**	59.1	4	4	60	F
A991	0.283	0.102	**0.181**	37.5	3	3	71	F
A992	0.378	0.199	**0.179**	37.1	4	4	57	F
A993	0.433	0.137	**0.296**	56.6	4	4	53	F
A994	0.486	0.106	**0.38**	70.6	4	4	64	M
A995	0.236	0.039	**0.197**	40.1	3	4	57	F
A996	0.166	0.019	**0.147**	31.8	3	3	52	F
A997	0.21	0.045	**0.165**	34.8	4	4	47	F
A998	0.221	0.081	**0.14**	30.7	3	3	55	F
A999	0.503	0.105	**0.398**	73.6	4	4	70	M
A1000	0.333	0.105	**0.228**	45.3	3	3	1y2m	M
A1001	0.394	0.082	**0.312**	59.3	3	4	50	F
A1002	0.455	0.287	**0.168**	35.3	3	3	31	F
A1003	0.374	0.074	**0.3**	57.3	3	4	50	M
A1004	0.22	0.062	**0.158**	33.7	3	4	21	F
A1005	0.164	0.01	**0.154**	33.0	4	4	73	M
A1006	0.516	0.137	**0.379**	70.4	5	5	47	F
A1007	0.555	0.185	**0.37**	68.9	4	4	24	M
A1008	0.47	0.087	**0.383**	71.1	5	5	45	F
A1009	0.516	0.082	**0.434**	79.6	5	5	36	F
A1010	0.659	0.085	**0.574**	102.9	5	5	68	M
A1011	0.679	0.07	**0.609**	108.7	6	6	72	F
A1012	0.598	0.064	**0.534**	96.2	5	5	51	F
A1013	0.32	0.046	**0.274**	53.0	4	4	19	M
A1014	0.425	0.121	**0.304**	57.9	4	4	6	F
A1015	0.347	0.074	**0.273**	52.8	4	4	62	F
A1016	0.49	0.07	**0.42**	77.2	4	4	56	M
A1017	0.502	0.071	**0.431**	79.1	4	4	42	F
A1018	0.598	0.068	**0.53**	95.5	5	5	38	F
