# Supplementary material for: Prevalence of G6PD deficiency in selected populations from two previously high malaria endemic areas of Sri Lanka
Source: PLoS One. 2017 Feb 2;12(2):e0171208. doi: 10.1371/journal.pone.0171208 (PMC5289554; doi:10.1371/journal.pone.0171208)
Supplement: S2 File — (PDF) [file pone.0171208.s002.pdf]

Sample	450nm	630nm	450-630nm	%	Colour O1	Colour O2	Sex	Age
**Plate 1**								
Blank	0	0	0		No	No		
Sub Negative	0.152	0.102	0.05		No	No		
Inactivated	0.322	0.29	0.032		no	No		
100	1.767	0.359	1.408		6	6		
60	0.971	0.152	0.819		6	6		
30	0.611	0.176	0.435		4	4		
10	0.43	0.175	0.255		3	3		
1	1.4	0.483	0.917	64.36	6	6	F	60
2	0.542	0.054	0.488	31.54	5	5	F	42
3	0.804	0.179	0.625	42.02	5	5	F	28
4	0.735	0.053	0.682	46.39	5	5	F	19
5	1.527	0.427	1.1	78.36	6	6	F	35
6	0.751	0.068	0.683	46.46	6	5	F	63
7	1.078	0.396	0.682	46.39	5	5	F	52
8	0.596	0.051	0.545	35.90	5	5	F	43
9	0.588	0.024	0.564	37.36	5	5	F	38
10	0.747	0.033	0.714	48.83	5	5	M	15
11	0.493	0.019	0.474	30.47	5	4	M	74
12	0.62	0.027	0.593	39.58	4	4	F	70
13	0.747	0.216	0.531	34.83	5	5	F	60
14	1.596	0.425	1.171	83.79	6	6	F	50
15	1.065	0.402	0.663	44.93	6	6	F	68
16	0.913	0.096	0.817	56.71	5	6	F	55
17	0.992	0.401	0.591	39.42	5	5	F	57
18	0.833	0.055	0.778	53.73	5	5	M	69
19	0.784	0.03	0.754	51.89	5	5	F	53
20	0.497	0.024	0.473	30.40	4	4	M	83
21	0.611	0.107	0.504	32.77	5	5	F	57
22	1.347	0.517	0.83	57.71	6	6	M	55
23	1.1	0.537	0.563	37.28	5	5	F	59
24	0.979	0.101	0.878	61.38	6	6	M	45
25	1.155	0.465	0.69	47.00	6	6	M	21
26	0.613	0.043	0.57	37.82	5	5	F	16
27	0.59	0.024	0.566	37.51	5	5	F	64
28	0.367	0.146	0.221	11.12	2	2	M	39
29	0.948	0.075	0.873	61.00	6	6	F	23
30	0.712	0.124	0.588	39.19	5	5	F	73
31	0.542	0.053	0.489	31.62	5	5	M	59
32	1.372	0.521	0.851	59.31	6	6	F	72
33	1.624	0.464	1.16	82.95	6	6	M	20
34	0.66	0.082	0.578	38.43	5	5	M	59

35	1.282	0.259	**1.023**	72.47	6	6	M	70
36	0.331	0.053	**0.278**	15.48	3	3	F	83
37	0.507	0.024	**0.483**	31.16	4	4	M	63
38	1.074	0.596	**0.478**	30.78	4	4	F	70
39	0.492	0.021	**0.471**	30.24	4	4	M	54
40	0.324	0.041	**0.283**	15.86	3	3	F	66
41	0.737	0.042	**0.695**	47.38	4	4	M	58
42	0.659	0.1	**0.559**	36.98	4	4	F	56
43	0.588	0.036	**0.552**	36.44	4	4	F	36
44	0.834	0.058	**0.776**	53.58	4	4	F	16
45	0.514	0.042	**0.472**	30.32	4	4	M	32
46	1.874	0.442	**1.432**	103.76	6	6	F	42
47	0.961	0.087	**0.874**	61.07	6	6	F	56
48	1.046	0.134	**0.912**	63.98	6	6	M	56
49	0.897	0.053	**0.844**	58.78	6	6	F	18
50	0.955	0.432	**0.523**	34.22	4	4	F	69
51	0.927	0.124	**0.803**	55.64	5	5	M	58
52	0.542	0.069	**0.473**	30.40	5	4	F	75
53	0.537	0.047	**0.49**	31.70	4	4	F	72
54	0.986	0.158	**0.828**	57.55	6	5	F	48
55	0.514	0.045	**0.469**	30.09	4	4	M	62
56	1.897	0.085	**1.812**	132.83	6	6	F	49
57	1.29	0.07	**1.22**	87.54	6	6	F	13
58	1.036	0.132	**0.904**	63.37	6	6	F	14
59	0.786	0.034	**0.752**	51.74	5	5	F	59
60	0.701	0.045	**0.656**	44.40	5	5	M	60
61	0.522	0.05	**0.472**	30.32	4	4	F	53
62	0.58	0.102	**0.478**	30.78	4	4	F	43
63	1.04	0.086	**0.954**	67.19	6	6	F	60
64	0.71	0.089	**0.621**	41.72	4	4	F	54
65	1.283	0.332	**0.951**	66.96	5	4	F	60
66	0.667	0.072	**0.595**	39.73	4	4	F	49
67	0.497	0.013	**0.484**	31.24	3	3	F	66
68	0.312	0.044	**0.268**	14.71	3	3	F	46
69	0.695	0.079	**0.616**	41.34	4	4	F	77
70	0.791	0.305	**0.486**	31.39	4	4	F	57
71	0.622	0.126	**0.496**	32.16	5	5	F	73
72	0.898	0.117	**0.781**	53.96	5	5	F	41
73	0.607	0.104	**0.503**	32.69	4	4	F	53
74	0.657	0.053	**0.604**	40.42	4	4	F	63
75	0.869	0.044	**0.825**	57.32	4	4	F	74
76	0.626	0.072	**0.554**	36.59	4	4	M	58
77	0.544	0.024	**0.52**	33.99	4	4	F	60

78	1.053	0.214	**0.839**	58.40	6	6	F	64
79	0.503	0.034	**0.469**	30.09	4	4	F	69
80	0.549	0.075	**0.474**	30.47	4	4	F	63
81	1.118	0.332	**0.786**	54.34	5	5	F	48
82	0.792	0.088	**0.704**	48.07	4	4	M	64
83	1.223	0.335	**0.888**	62.14	5	5	M	31
84	0.883	0.042	**0.841**	58.55	5	5	F	56
**Plate 2**								
Blank	**0.023**	**0**	**0.023**		**No**	**No**		
Sub Negative	**0.139**	**0.101**	**0.038**		**NO**	**No**		
Inactivated	**0.144**	**0.113**	**0.031**		**no**	**No**		
100	**1.148**	**0.059**	**1.089**		**6**	**6**		
60	**0.785**	**0.091**	**0.694**		**4**	**4**		
30	**0.385**	**0.064**	**0.321**		**3**	**3**		
10	**0.374**	**0.085**	**0.289**		**3**	**3**		
85	0.744	0.057	**0.687**	59.12	5	5	F	64
86	1.064	0.539	**0.525**	42.47	5	5	F	63
87	1.316	0.461	**0.855**	76.38	5	5	F	14
88	0.369	0.051	**0.318**	21.21	3	3	M	58
89	0.68	0.156	**0.524**	42.37	4	4	M	40
90	0.456	0.014	**0.442**	33.95	3	3	F	34
91	0.42	0.012	**0.408**	30.45	3	3	F	60
92	0.354	0.119	**0.235**	12.68	3	3	F	74
93	0.889	0.471	**0.418**	31.48	4	4	M	69
94	0.508	0.018	**0.49**	38.88	3	3	F	68
95	0.743	0.078	**0.665**	56.86	5	5	F	62
96	0.49	0.019	**0.471**	36.93	4	4	F	55
97	0.66	0.121	**0.539**	43.91	5	4	F	60
98	0.812	0.126	**0.686**	59.02	5	4	F	38
99	0.628	0.071	**0.557**	45.76	5	4	F	53
100	0.614	0.054	**0.56**	46.07	4	4	F	47
101	0.315	0.022	**0.293**	18.64	3	3	F	50
102	0.825	0.23	**0.595**	49.67	5	5	F	52
103	1.714	0.164	**1.55**	147.79	6	6	F	43
104	1.337	0.062	**1.275**	119.53	6	6	M	59
105	0.537	0.109	**0.428**	32.51	4	4	F	60
106	0.997	0.137	**0.86**	76.89	4	4	F	44
107	1.072	0.047	**1.025**	93.85	4	4	F	44
108	0.473	0.053	**0.42**	31.69	3	3	F	57
109	0.669	0.054	**0.615**	51.72	3	3	F	55
110	1.405	0.097	**1.308**	122.92	5	5	F	42
111	1.848	0.456	**1.392**	131.55	5	5	F	56
112	0.518	0.085	**0.433**	33.02	4	4	F	60

113	1.558	0.254	**1.304**	122.51	5	5	F	23
114	0.805	0.08	**0.725**	63.02	4	4	F	31
115	0.535	0.028	**0.507**	40.62	4	4	F	54
116	0.82	0.261	**0.559**	45.97	4	4	M	66
117	0.888	0.459	**0.429**	32.61	4	4	F	63
118	0.613	0.061	**0.552**	45.25	4	4	F	47
119	0.423	0.016	**0.407**	30.35	4	4	F	3y6m
120	0.836	0.171	**0.665**	56.86	4	5	F	52
121	0.693	0.096	**0.597**	49.87	5	5	F	64
122	1.367	0.081	**1.286**	120.66	6	6	M	79
123	0.765	0.044	**0.721**	62.61	5	5	M	65
124	0.98	0.055	**0.925**	83.57	5	5	F	53
125	0.834	0.083	**0.751**	65.69	5	5	F	35
126	1.624	0.125	**1.499**	142.55	6	6	F	58
127	0.736	0.033	**0.703**	60.76	5	5	F	34
128	0.495	0.037	**0.458**	35.59	5	5	F	42
129	0.779	0.152	**0.627**	52.95	5	5	F	47
130	0.51	0.086	**0.424**	32.10	5	4	F	70
131	0.852	0.073	**0.779**	68.57	5	5	F	53
132	0.952	0.082	**0.87**	77.92	5	5	F	70
133	0.889	0.073	**0.816**	72.37	5	5	F	60
134	0.901	0.116	**0.785**	69.19	5	5	F	45
135	0.65	0.028	**0.622**	52.44	5	5	F	76
136	0.783	0.025	**0.758**	66.41	5	5	F	63
137	0.429	0.022	**0.407**	30.35	4	3	F	63
138	0.343	0.029	**0.314**	20.80	3	2	F	75
139	0.476	0.065	**0.411**	30.76	3	3	F	68
140	0.376	0.112	**0.264**	15.66	2	2	F	51
141	0.73	0.088	**0.642**	54.50	4	4	F	68
142	0.586	0.064	**0.522**	42.17	4	4	M	67
143	1.37	0.487	**0.883**	79.26	4	4	F	64
144	1.016	0.439	**0.577**	47.82	4	4	F	58
145	0.666	0.066	**0.6**	50.18	4	4	F	50
146	0.508	0.102	**0.406**	30.25	3	3	F	69
147	0.967	0.144	**0.823**	73.09	4	4	F	72
148	0.446	0.026	**0.42**	31.69	3	3	F	67
149	0.896	0.081	**0.815**	72.27	4	4	F	53
150	1.244	0.078	**1.166**	108.33	6	6	F	45
151	0.845	0.056	**0.789**	69.60	5	5	M	56
152	1.111	0.35	**0.761**	66.72	5	5	F	70
153	0.785	0.107	**0.678**	58.19	5	5	F	63
154	0.869	0.128	**0.741**	64.67	4	4	F	54
155	1.233	0.494	**0.739**	64.46	4	4	M	47

156	0.751	0.092	**0.659**	56.24	4	4	F	52
157	0.942	0.055	**0.887**	79.67	4	4	F	77
158	0.49	0.036	**0.454**	35.18	4	4	F	20
159	1.316	0.455	**0.861**	77.00	4	4	F	20
160	0.577	0.077	**0.5**	39.91	4	4	F	52
161	0.706	0.069	**0.637**	53.98	4	4	M	56
162	0.746	0.09	**0.656**	55.93	4	4	F	34
163	1.159	0.067	**1.092**	100.73	5	5	F	43
164	1.399	0.288	**1.111**	102.68	5	5	F	55
165	0.95	0.06	**0.89**	79.98	5	5	M	35
166	0.883	0.06	**0.823**	73.09	5	5	F	65
167	0.315	0.055	**0.26**	15.25	3	3	F	47
168	0.746	0.05	**0.696**	60.04	4	4	F	49
**Plate 3**								
Blank	**0**	**0**	**0**		**No**	**No**		
Sub Negative	**0.061**	**0.043**	**0.018**		**No**	**No**		
Inactivated	**0.095**	**0.071**	**0.024**		**No**	**No**		
100	**1.221**	**0.043**	**1.178**		**5**	**5**		
60	**0.832**	**0.05**	**0.782**		**4**	**4**		
30	**0.513**	**0.158**	**0.355**		**3**	**3**		
10	**0.321**	**0.067**	**0.254**		**2**	**2**		
169	1.41	0.369	**1.041**	86.54	5	5	M	63
170	0.439	0.009	**0.43**	30.55	4	4	M	76
171	1.036	0.041	**0.995**	82.32	5	5	M	50
172	0.618	0.033	**0.585**	44.75	5	4	F	52
173	0.775	0.171	**0.604**	46.50	4	4	F	62
174	0.685	0.053	**0.632**	49.06	4	4	F	54
175	0.54	0.037	**0.503**	37.24	4	4	F	61
176	0.962	0.232	**0.73**	58.04	4	4	M	50
177	0.908	0.021	**0.887**	72.43	4	4	M	64
178	0.775	0.304	**0.471**	34.31	4	4	F	49
179	0.793	0.112	**0.681**	53.55	4	4	F	58
180	0.364	0.109	**0.255**	14.52	2	2	F	72
181	0.721	0.062	**0.659**	51.53	4	4	F	52
182	0.527	0.067	**0.46**	33.30	4	4	F	60
183	0.456	0.026	**0.43**	30.55	4	4	F	63
184	0.196	0.027	**0.169**	6.64	3	3	F	63
185	0.533	0.042	**0.491**	36.14	3	3	M	75
186	0.991	0.036	**0.955**	78.66	5	5	M	50
187	0.753	0.052	**0.701**	55.38	5	5	M	73
188	0.448	0.018	**0.43**	30.55	4	4	F	39
189	0.987	0.201	**0.786**	63.17	6	6	F	77
190	1.476	0.37	**1.106**	92.49	5	5	F	56

191	0.921	0.06	**0.861**	70.04	5	5	F	53
192	0.943	0.294	**0.649**	50.62	4	4	F	51
193	1.197	0.561	**0.636**	49.43	4	4	F	54
194	1.11	0.047	**1.063**	88.55	5	5	F	49
195	0.811	0.033	**0.778**	62.44	5	5	F	53
196	1.428	0.122	**1.306**	110.82	6	6	F	36
197	0.691	0.035	**0.656**	51.26	4	4	F	63
198	0.595	0.066	**0.529**	39.62	3	3	F	64
199	0.993	0.087	**0.906**	74.17	5	5	F	48
200	0.74	0.053	**0.687**	54.10	4	4	F	64
201	1.054	0.129	**0.925**	75.91	4	4	F	70
202	0.562	0.072	**0.49**	36.05	3	3	M	63
203	0.813	0.051	**0.762**	60.97	4	4	F	55
204	0.763	0.055	**0.708**	56.02	4	4	M	74
205	0.515	0.047	**0.468**	34.03	2	3	F	67
206	0.455	0.022	**0.433**	30.83	3	3	F	55
207	0.815	0.051	**0.764**	61.16	4	4	F	54
208	0.458	0.014	**0.444**	31.84	3	3	F	57
209	0.515	0.036	**0.479**	35.04	3	3	F	51
210	0.675	0.036	**0.639**	49.70	3	3	F	48
211	0.583	0.016	**0.567**	43.11	3	3	M	66
212	0.671	0.034	**0.637**	49.52	3	4	F	63
213	0.566	0.115	**0.451**	32.48	3	3	F	64
214	0.855	0.085	**0.77**	61.71	5	5	F	62
215	0.994	0.477	**0.517**	38.52	4	4	F	40
216	0.733	0.06	**0.673**	52.82	4	4	M	52
217	0.674	0.161	**0.513**	38.16	4	4	F	65
218	0.572	0.121	**0.451**	32.48	4	4	F	62
219	0.531	0.102	**0.429**	30.46	3	3	F	56
220	0.811	0.297	**0.514**	38.25	4	4	F	46
221	1.185	0.186	**0.999**	82.69	5	5	M	56
222	0.95	0.122	**0.828**	67.02	5	5	M	38
223	0.722	0.079	**0.643**	50.07	5	5	M	64
224	0.892	0.273	**0.619**	47.87	5	5	F	59
225	0.441	0.01	**0.431**	30.64	3	3	F	70
226	0.781	0.078	**0.703**	55.57	5	5	M	38
227	0.599	0.165	**0.434**	30.92	3	3	F	70
228	0.755	0.161	**0.594**	45.58	4	4	M	45
229	0.518	0.083	**0.435**	31.01	3	3	F	55
230	0.483	0.051	**0.432**	30.74	3	3	F	56
231	0.501	0.07	**0.431**	30.64	3	3	M	58
232	1.212	0.422	**0.79**	63.54	4	4	F	53
233	1.112	0.4	**0.712**	56.39	4	4	F	55

234	0.682	0.079	**0.603**	46.40	4	4	F	50
235	0.505	0.06	**0.445**	31.93	3	3	M	72
236	0.439	0.013	**0.426**	30.19	3	3	M	80
237	0.793	0.109	**0.684**	53.83	4	4	F	46
238	0.77	0.153	**0.617**	47.69	3	3	F	60
239	1.072	0.114	**0.958**	78.93	4	4	F	62
240	0.602	0.088	**0.514**	38.25	3	3	F	56
241	0.719	0.07	**0.649**	50.62	4	4	F	64
242	1.062	0.104	**0.958**	78.93	5	5	M	43
243	0.92	0.133	**0.787**	63.26	4	4	F	63
244	0.753	0.123	**0.63**	48.88	4	4	F	68
245	0.843	0.07	**0.773**	61.98	5	5	F	28
246	0.539	0.104	**0.435**	31.01	4	4	M	70
247	0.579	0.093	**0.486**	35.68	4	4	F	55
248	1.025	0.119	**0.906**	74.17	5	5	M	39
249	0.708	0.099	**0.609**	46.95	4	4	F	48
250	0.649	0.05	**0.599**	46.04	4	4	F	22
251	0.544	0.048	**0.496**	36.60	4	4	F	46
252	0.436	0.005	**0.431**	30.64	4	4	M	53
**Plate 4**								
Blank	**0**	**0**	**0**		**No**	**No**		
Sub Negative	**0.085**	**0.06**	**0.025**		**No**	**NO**		
Inactivated	**0.157**	**0.238**	**-0.081**		**No**	**No**		
100	**1.414**	**0.041**	**1.373**		**5**	**5**		
60	**0.915**	**0.09**	**0.825**		**5**	**5**		
30	**0.486**	**0.088**	**0.398**		**2**	**2**		
10	**0.324**	**0.056**	**0.268**		**2**	**2**		
253	0.302	0.024	**0.278**	15.79	2	2	F	68
254	0.531	0.051	**0.48**	31.57	3	3	F	55
255	0.768	0.131	**0.637**	43.83	3	3	F	63
256	0.518	0.011	**0.507**	33.67	3	3	F	71
257	1.046	0.099	**0.947**	68.04	5	6	F	41
258	0.676	0.169	**0.507**	33.67	4	4	F	25
259	0.756	0.272	**0.484**	31.88	4	4	F	62
260	0.675	0.162	**0.513**	34.14	4	4	F	38
261	0.646	0.148	**0.498**	32.97	4	4	F	70
262	0.213	0.012	**0.201**	9.77	3	3	M	59
263	0.295	0.029	**0.266**	14.85	3	3	M	56
264	0.84	0.038	**0.802**	56.72	4	4	M	44
265	0.494	0.024	**0.47**	30.78	4	4	F	41
266	1.26	0.177	**1.083**	78.67	5	5	F	51
267	0.604	0.039	**0.565**	38.21	4	4	F	41
268	0.747	0.059	**0.688**	47.81	4	4	F	47

269	0.585	0.033	**0.552**	37.19	4	4	F	38
270	0.922	0.092	**0.83**	58.90	5	5	F	50
271	0.771	0.027	**0.744**	52.19	4	4	F	58
272	0.546	0.04	**0.506**	33.60	4	4	F	52
273	0.976	0.312	**0.664**	45.94	4	4	F	65
274	0.88	0.194	**0.686**	47.66	5	5	F	38
275	0.677	0.064	**0.613**	41.95	4	4	F	50
276	0.767	0.055	**0.712**	49.69	5	5	M	44
277	0.84	0.044	**0.796**	56.25	4	4	F	33
278	0.359	0.063	**0.296**	17.19	3	3	M	55
279	0.476	0.011	**0.465**	30.39	3	3	F	48
280	0.988	0.243	**0.745**	52.27	4	4	F	22
281	0.884	0.145	**0.739**	51.80	4	4	F	44
282	0.92	0.183	**0.737**	51.64	5	5	M	72
283	0.498	0.032	**0.466**	30.47	3	5	M	58
284	0.774	0.092	**0.682**	47.34	5	5	F	39
285	0.851	0.078	**0.773**	54.45	4	4	M	61
286	0.198	0.087	**0.111**	2.74	3	3	M	63
287	0.987	0.262	**0.725**	50.70	3	4	M	58
288	0.574	0.012	**0.562**	37.97	3	3	F	52
**Plate 5**								
Blank	**0**	**0**	**0**		**No**	**No**		
Sub Negative	**0.109**	**0.084**	**0.025**		**No**	**No**		
Inactivated	**0.241**	**0.202**	**0.039**		**No**	**No**		
100	**1.419**	**0.04**	**1.379**		**5**	**5**		
60	**1.049**	**0.25**	**0.799**		**5**	**5**		
30	**0.648**	**0.23**	**0.418**		**3**	**3**		
10	**0.352**	**0.057**	**0.295**		**2**	**2**		
289	0.59	0.012	**0.578**	38.46	2	3	F	54
290	0.497	0.015	**0.482**	30.81	2	3	F	53
291	0.499	0.014	**0.485**	31.05	3	4	M	30
292	0.783	0.016	**0.767**	53.53	3	4	F	54
293	0.789	0.011	**0.778**	54.40	3	3	F	57
294	0.509	0.01	**0.499**	32.16	3	3	F	38
295	0.796	0.044	**0.752**	52.33	3	3	F	66
296	0.521	0.026	**0.495**	31.85	3	3	F	65
297	0.552	0.033	**0.519**	33.76	3	3	F	64
298	0.632	0.012	**0.62**	41.81	3	3	F	60
299	0.719	0.033	**0.686**	47.07	4	4	F	47
300	0.909	0.023	**0.886**	63.01	4	4	F	53
301	0.936	0.32	**0.616**	41.49	4	4	F	65
302	0.746	0.089	**0.657**	44.76	2	2	F	72
303	0.699	0.102	**0.597**	39.98	2	2	F	68

304	0.722	0.011	**0.711**	49.06	5	5	F	70
305	0.56	0.045	**0.515**	33.44	2	2	F	69
306	0.289	0.016	**0.273**	14.15	2	2	F	71
307	0.635	0.024	**0.611**	41.09	3	3	F	35
308	0.641	0.013	**0.628**	42.45	4	3	F	52
309	0.638	0.014	**0.624**	42.13	3	3	F	35
310	0.299	0.012	**0.287**	15.27	2	2	F	65
311	0.329	0.015	**0.314**	17.42	2	2	F	54
312	1.176	0.045	**1.131**	82.54	5	5	F	47
313	0.215	0.004	**0.211**	9.21	2	2	M	52
314	0.495	0.015	**0.48**	30.65	3	3	M	50
315	1.013	0.018	**0.995**	71.70	4	4	F	63
316	0.868	0.167	**0.701**	48.27	4	4	F	58
317	0.802	0.019	**0.783**	54.80	4	4	M	60
318	0.577	0.017	**0.56**	37.03	4	4	M	55
319	0.49	0.012	**0.478**	30.49	3	3	M	49
320	0.596	0.026	**0.57**	37.82	4	4	F	70
321	0.591	0.014	**0.577**	38.38	4	4	M	63
322	0.527	0.02	**0.507**	32.80	4	4	F	64
323	0.57	0.024	**0.546**	35.91	4	4	F	60
324	0.227	0.007	**0.22**	9.93	2	2	F	60
325	0.633	0.147	**0.486**	31.13	3	3	M	62
326	0.654	0.049	**0.605**	40.61	3	3	F	57
327	0.164	0.012	**0.152**	4.50	2	2	F	70
328	0.283	0.01	**0.273**	14.15	2	2	F	34
329	0.611	0.042	**0.569**	37.74	4	4	M	63
330	0.301	0.015	**0.286**	15.19	3	3	F	45
331	0.685	0.011	**0.674**	46.11	5	4	F	72
332	0.649	0.016	**0.633**	42.85	5	5	F	63
333	0.551	0.024	**0.527**	34.40	3	3	M	54
334	0.61	0.074	**0.536**	35.11	4	4	M	58
335	0.911	0.018	**0.893**	63.57	5	5	M	72
336	0.795	0.111	**0.684**	46.91	5	5	M	72
337	0.491	0.015	**0.476**	30.33	4	4	F	65
338	0.229	0.018	**0.211**	9.21	2	2	F	58
339	0.779	0.016	**0.763**	53.21	4	4	F	57
340	0.953	0.146	**0.807**	56.72	4	4	F	31
341	0.709	0.025	**0.684**	46.91	4	4	F	51
342	0.661	0.181	**0.48**	30.65	3	3	F	52
343	0.689	0.043	**0.646**	43.88	5	5	M	66
344	0.674	0.032	**0.642**	43.56	5	5	F	63
345	0.501	0.016	**0.485**	31.05	4	4	M	60
346	0.651	0.026	**0.625**	42.21	5	5	F	54

347	0.3	0.008	**0.292**	15.66	3	3	F	75
348	0.27	0.04	**0.23**	10.72	3	3	F	49
349	0.589	0.052	**0.537**	35.19	4	4	F	63
350	0.521	0.037	**0.484**	30.97	3	3	F	63
351	0.392	0.05	**0.342**	19.65	3	3	F	63
352	0.56	0.06	**0.5**	32.24	3	3	F	73
353	0.342	0.053	**0.289**	15.43	3	3	F	52
354	0.489	0.011	**0.478**	30.49	4	4	F	63
355	0.948	0.109	**0.839**	59.27	4	5	F	46
356	0.276	0.018	**0.258**	12.95	2	2	F	39
357	0.682	0.055	**0.627**	42.37	4	4	F	73
358	0.603	0.099	**0.504**	32.56	3	3	F	56
359	0.523	0.041	**0.482**	30.81	3	3	F	58
360	0.495	0.016	**0.479**	30.57	3	3	F	62
361	0.724	0.067	**0.657**	44.76	4	4	M	53
362	0.567	0.09	**0.477**	30.41	4	4	F	73
363	0.63	0.035	**0.595**	39.82	4	4	F	38
364	0.518	0.04	**0.478**	30.49	4	4	F	57
365	1.008	0.091	**0.917**	65.48	5	5	F	64
366	0.487	0.006	**0.481**	30.73	3	3	F	72
367	0.572	0.07	**0.502**	32.40	3	3	F	53
368	0.342	0.113	**0.229**	10.64	3	3	F	70
369	0.805	0.061	**0.744**	51.69	5	5	F	56
370	0.584	0.073	**0.511**	33.12	5	5	M	76
371	0.543	0.066	**0.477**	30.41	4	4	F	53
372	0.638	0.055	**0.583**	38.86	4	4	F	57
**Plate 6**								
Blank	**0**	**0**	**0**		**No**	**No**		
Sub Negative	**0.074**	**0.068**	**0.006**		**No**	**No**		
Inactivated	**0.044**	**0.021**	**0.023**		**No**	**No**		
100	**1.278**	**0.096**	**1.182**		**6**	**6**		
60	**0.612**	**0.027**	**0.585**		**3**	**3**		
30	**0.379**	**0.06**	**0.319**		**3**	**3**		
10	**0.265**	**0.02**	**0.245**		**2**	**2**		
373	0.523	0.024	**0.499**	42.47	3	3	M	40
374	0.364	0.055	**0.309**	25.40	2	2	F	68
375	0.264	0.008	**0.256**	20.64	2	2	F	63
376	0.286	0.052	**0.234**	18.66	3	3	F	52
377	0.389	0.016	**0.373**	31.15	3	3	F	32
378	0.966	0.066	**0.9**	78.51	5	5	M	40
379	0.758	0.165	**0.593**	50.92	3	3	M	40
380	0.591	0.08	**0.511**	43.55	3	3	F	70
381	0.807	0.057	**0.75**	65.03	4	4	F	51

382	0.619	0.03	**0.589**	50.56	3	3	F	66
383	0.428	0.007	**0.421**	35.47	3	3	F	49
384	0.294	0.041	**0.253**	20.37	2	2	F	59
385	0.519	0.112	**0.407**	34.21	3	3	F	75
386	0.577	0.083	**0.494**	42.03	3	3	F	51
387	1.136	0.094	**1.042**	91.27	6	6	M	24
388	0.655	0.034	**0.621**	53.44	3	3	F	48
389	0.561	0.051	**0.51**	43.46	3	3	F	56
390	0.433	0.043	**0.39**	32.68	2	2	F	74
391	0.421	0.025	**0.396**	33.22	3	3	F	60
392	0.811	0.019	**0.792**	68.80	4	4	M	40
393	0.354	0.009	**0.345**	28.64	3	3	M	58
394	0.667	0.031	**0.636**	54.78	4	4	M	52
395	0.807	0.261	**0.546**	46.70	5	5	F	73
396	0.699	0.066	**0.633**	54.52	3	3	F	9
397	0.433	0.027	**0.406**	34.12	2	2	M	11
398	0.88	0.062	**0.818**	71.14	5	5	F	7
399	0.234	0.13	**0.104**	6.98	2	2	F	55
400	0.723	0.059	**0.664**	57.30	4	4	F	47
401	0.798	0.079	**0.719**	62.24	5	5	M	13
402	0.69	0.208	**0.482**	40.95	4	4	M	1
403	1.291	0.059	**1.232**	108.34	6	6	F	7y 8m
404	0.749	0.37	**0.379**	31.69	3	3	F	70
405	0.631	0.021	**0.61**	52.45	4	3	F	44
406	1.473	0.385	**1.088**	95.40	5	5	F	39
407	1.125	0.42	**0.705**	60.99	4	4	M	58
408	1.314	0.167	**1.147**	100.70	5	5	F	60
409	1.027	0.047	**0.98**	85.70	5	5	M	77
410	0.978	0.142	**0.836**	72.76	5	5	M	58
411	0.909	0.096	**0.813**	70.69	5	5	F	54
412	0.8	0.05	**0.75**	65.03	5	5	F	58
413	1.052	0.288	**0.764**	66.29	4	4	M	46
414	1.072	0.393	**0.679**	58.65	4	4	M	53
415	1.097	0.337	**0.76**	65.93	4	4	F	53
416	0.811	0.103	**0.708**	61.25	4	4	F	11
417	1.175	0.27	**0.905**	78.96	5	5	M	67
418	1.592	0.58	**1.012**	88.57	5	5	F	60
419	1.185	0.265	**0.92**	80.30	5	5	M	66
420	0.847	0.054	**0.793**	68.89	5	5	M	44
421	0.774	0.051	**0.723**	62.60	4	4	F	27
422	0.73	0.059	**0.671**	57.93	4	4	M	14
423	1.254	0.082	**1.172**	102.95	5	5	F	14
424	0.642	0.069	**0.573**	49.12	4	4	F	17

425	0.992	0.255	**0.737**	63.86	4	4	F	33
426	0.802	0.1	**0.702**	60.72	4	4	F	72
427	0.88	0.049	**0.831**	72.31	3	4	F	80
428	1.246	0.081	**1.165**	102.32	6	6	F	43
429	0.741	0.041	**0.7**	60.54	4	4	F	77
430	0.824	0.106	**0.718**	62.15	4	4	F	59
431	1.287	0.083	**1.204**	105.82	5	5	F	50
432	0.902	0.028	**0.874**	76.17	5	5	F	33
433	1.245	0.099	**1.146**	100.61	5	5	M	52
434	1.096	0.424	**0.672**	58.02	4	4	F	57
435	1.576	0.464	**1.112**	97.56	5	5	F	65
436	1.044	0.091	**0.953**	83.27	5	5	F	38
437	0.68	0.082	**0.598**	51.37	4	3	F	80
438	1.397	0.515	**0.882**	76.89	4	4	M	62
439	0.981	0.042	**0.939**	82.01	4	4	F	55
440	0.778	0.354	**0.424**	35.74	4	4	M	63
441	1.037	0.459	**0.578**	49.57	4	4	F	43
442	0.817	0.129	**0.688**	59.46	4	4	M	72
443	0.949	0.192	**0.757**	65.66	5	5	F	37
444	0.86	0.112	**0.748**	64.85	4	4	F	78
445	1.338	0.086	**1.252**	110.14	5	5	F	54
446	1.218	0.078	**1.14**	100.07	5	5	F	51
447	1.16	0.054	**1.106**	97.02	5	5	M	68
448	1.016	0.272	**0.744**	64.49	4	4	F	56
449	0.785	0.062	**0.723**	62.60	4	4	F	54
450	0.755	0.123	**0.632**	54.43	4	4	F	62
451	0.402	0.034	**0.368**	30.70	2	2	F	61
452	0.878	0.11	**0.768**	66.65	4	4	F	52
453	0.894	0.071	**0.823**	71.59	5	5	F	48
454	0.784	0.076	**0.708**	61.25	4	4	M	60
455	0.684	0.077	**0.607**	52.18	4	4	M	62
456	0.575	0.057	**0.518**	44.18	4	4	F	42
**Plate 7**								
Blank	**0**	**0**	**0**		**No**	**No**		
Sub Negative	**0.112**	**0.084**	**0.028**		**No**	**No**		
Inactivated	**0.104**	**0.079**	**0.025**		**No**	**No**		
100	**1.474**	**0.224**	**1.25**		**5**	**5**		
60	**0.83**	**0.097**	**0.733**		**5**	**5**		
30	**0.519**	**0.072**	**0.447**		**3**	**3**		
10	**0.394**	**0.119**	**0.275**		**2**	**2**		
457	0.677	0.068	**0.609**	43.85	3	3	F	66
458	0.529	0.06	**0.469**	31.06	3	3	F	64
459	0.819	0.047	**0.772**	58.75	4	4	M	66

460	0.95	0.175	**0.775**	59.02	4	4	M	69
461	0.773	0.036	**0.737**	55.55	3	3	F	58
462	0.602	0.077	**0.525**	36.18	3	3	F	48
463	1.025	0.284	**0.741**	55.92	4	4	M	58
464	0.482	0.015	**0.467**	30.88	3	3	F	60
465	0.539	0.062	**0.477**	31.79	3	3	F	61
466	0.666	0.06	**0.606**	43.58	4	4	F	54
467	0.61	0.03	**0.58**	41.20	3	3	F	60
468	0.57	0.035	**0.535**	37.09	3	3	M	71
469	0.368	0.023	**0.345**	19.73	2	2	F	70
470	0.583	0.062	**0.521**	35.81	3	3	M	72
471	0.572	0.047	**0.525**	36.18	4	4	F	58
472	0.855	0.032	**0.823**	63.41	4	4	F	52
473	0.686	0.032	**0.654**	47.97	3	3	F	64
474	0.736	0.043	**0.693**	51.53	3	3	F	32
475	0.885	0.053	**0.832**	64.23	3	3	F	49
476	0.763	0.039	**0.724**	54.36	3	3	F	12
477	0.667	0.078	**0.589**	42.03	4	4	F	59
478	0.613	0.072	**0.541**	37.64	3	3	M	52
479	0.217	0.07	**0.147**	1.64	2	2	M	64
480	0.78	0.133	**0.647**	47.33	4	4	F	62
481	0.821	0.027	**0.794**	60.76	4	4	F	37
482	0.675	0.064	**0.611**	44.04	4	4	F	46
483	0.574	0.035	**0.539**	37.46	4	4	F	56
484	0.501	0.018	**0.483**	32.34	3	3	F	52
485	0.646	0.045	**0.601**	43.12	4	4	F	70
486	0.854	0.07	**0.784**	59.85	4	4	M	52
487	0.743	0.054	**0.689**	51.17	4	4	F	71
488	0.402	0.037	**0.365**	21.56	2	2	F	35
489	0.682	0.025	**0.657**	48.24	4	4	M	5y6m
490	0.575	0.041	**0.534**	37.00	3	3	F	69
491	0.92	0.023	**0.897**	70.17	4	4	M	6
492	0.777	0.027	**0.75**	56.74	4	4	M	65
493	0.479	0.012	**0.467**	30.88	3	3	F	37
494	0.693	0.188	**0.505**	34.35	3	3	F	39
495	0.684	0.036	**0.648**	47.42	4	4	F	48
496	0.405	0.088	**0.317**	17.17	2	2	F	52
497	0.605	0.058	**0.547**	38.19	3	3	F	42
498	0.571	0.078	**0.493**	33.25	3	2	F	46
499	0.402	0.016	**0.386**	23.48	2	2	M	46
500	0.764	0.034	**0.73**	54.91	4	4	F	36
501	0.826	0.061	**0.765**	58.11	4	4	M	56
502	0.616	0.072	**0.544**	37.92	3	3	F	60

503	0.862	0.058	**0.804**	61.67	4	4	M	78
504	0.922	0.113	**0.809**	62.13	5	5	M	62
505	1.115	0.323	**0.792**	60.58	5	5	F	58
506	0.426	0.054	**0.372**	22.20	2	2	F	60
507	0.5	0.025	**0.475**	31.61	2	2	M	59
508	0.162	0.022	**0.14**	1.00	2	2	M	65
509	0.197	0.048	**0.149**	1.82	1	1	F	25
510	0.681	0.094	**0.587**	41.84	4	4	M	55
511	0.629	0.061	**0.568**	40.11	4	4	F	41
512	0.763	0.07	**0.693**	51.53	4	4	F	43
513	0.479	0.013	**0.466**	30.79	3	3	F	54
514	0.907	0.05	**0.857**	66.52	4	4	F	66
515	0.92	0.041	**0.879**	68.53	4	4	M	65
516	0.614	0.055	**0.559**	39.29	4	4	F	66
517	0.558	0.065	**0.493**	33.25	3	3	F	63
518	0.684	0.103	**0.581**	41.30	3	4	F	63
519	0.712	0.073	**0.639**	46.60	4	4	F	29
520	0.629	0.113	**0.516**	35.36	4	4	F	65
521	0.747	0.077	**0.67**	49.43	4	4	F	42
522	0.569	0.064	**0.505**	34.35	3	3	M	65
523	0.513	0.047	**0.466**	30.79	3	3	M	63
524	0.711	0.064	**0.647**	47.33	3	3	M	44
525	0.344	0.08	**0.264**	12.33	3	3	M	57
526	0.626	0.106	**0.52**	35.72	3	3	F	62
527	0.59	0.087	**0.503**	34.17	3	3	M	47
528	0.755	0.093	**0.662**	48.70	3	2	F	51
529	0.704	0.092	**0.612**	44.13	3	3	F	34
530	0.546	0.079	**0.467**	30.88	3	3	F	52
531	0.341	0.057	**0.284**	14.16	2	2	F	57
532	0.758	0.063	**0.695**	51.71	3	3	F	32
533	0.606	0.124	**0.482**	32.25	3	3	F	43
534	0.786	0.103	**0.683**	50.62	4	4	F	65
535	0.694	0.103	**0.591**	42.21	4	4	M	66
536	1.08	0.123	**0.957**	75.65	4	4	M	74
537	0.716	0.1	**0.616**	44.49	4	4	F	50
538	0.535	0.06	**0.475**	31.61	3	3	F	48
539	0.396	0.073	**0.323**	17.72	2	2	M	63
540	0.216	0.056	**0.16**	2.83	1	1	F	79
**Plate 8**								
Blank	**0**	**0**	**0**		**No**	**No**		
Sub Negative	**0.167**	**0.13**	**0.037**		**No**	**No**		
Inactivated	**0.205**	**0.141**	**0.064**		**No**	**no**		
100	**1.074**	**0.224**	**0.85**		**5**	**5**		

60	**0.672**	**0.057**	**0.615**		**5**	**5**		
30	**0.429**	**0.052**	**0.377**		**3**	**3**		
10	**0.374**	**0.119**	**0.255**		**2**	**2**		
541	0.48	0.082	**0.398**	31.29	2	2	F	47
542	0.494	0.08	**0.414**	33.66	2	2	F	53
543	0.448	0.052	**0.396**	30.99	2	2	F	57
544	0.676	0.086	**0.59**	59.75	3	3	F	54
545	0.243	0.033	**0.21**	3.42	2	2	M	53
546	0.487	0.077	**0.41**	33.06	3	3	M	72
547	0.673	0.061	**0.612**	63.01	4	4	M	65
548	0.588	0.094	**0.494**	45.52	4	4	F	50
549	0.419	0.022	**0.397**	31.14	2	2	F	66
550	0.468	0.07	**0.398**	31.29	3	3	F	56
551	0.702	0.044	**0.658**	69.83	4	4	M	52
552	0.508	0.053	**0.455**	39.74	3	3	F	48
553	0.587	0.036	**0.551**	53.97	3	3	F	22
554	0.503	0.039	**0.464**	41.07	3	3	M	75
555	0.409	0.016	**0.393**	30.54	3	3	F	42
556	0.548	0.069	**0.479**	43.29	3	3	F	66
557	0.594	0.098	**0.496**	45.81	3	3	F	54
558	0.756	0.08	**0.676**	72.49	4	4	F	60
559	0.419	0.029	**0.39**	30.10	3	3	F	68
560	0.559	0.061	**0.498**	46.11	3	3	F	56
561	0.472	0.042	**0.43**	36.03	3	3	F	58
562	0.443	0.046	**0.397**	31.14	3	3	F	80
563	0.722	0.027	**0.695**	75.31	4\3	4	F	59
564	0.965	0.076	**0.889**	104.07	4	4	F	25
565	0.766	0.073	**0.693**	75.01	4	4	F	59
566	1.157	0.36	**0.797**	90.43	4	4	F	62
567	0.507	0.042	**0.465**	41.22	3	3	F	68
568	0.822	0.059	**0.763**	85.39	4	4	M	58
569	0.548	0.052	**0.496**	45.81	3	3	M	72
570	0.358	0.05	**0.308**	17.95	2\3	2	F	65
571	0.553	0.053	**0.5**	46.41	3	3	F	49
572	0.48	0.083	**0.397**	31.14	3	3	F	60
573	0.671	0.055	**0.616**	63.60	4	4	M	66
574	0.727	0.059	**0.668**	71.31	4	4	F	63
575	0.456	0.031	**0.425**	35.29	3	3	M	64
576	0.27	0.039	**0.231**	6.53	2	2	F	64
577	0.604	0.021	**0.583**	58.71	4	4	F	46
578	0.319	0.034	**0.285**	14.54	3	3	F	54
579	0.69	0.076	**0.614**	63.30	4\3	3	M	72
580	0.48	0.071	**0.409**	32.92	3	3	F	41

581	0.497	0.064	**0.433**	36.47	3	3	M	68
582	0.474	0.058	**0.416**	33.95	3	3	F	60
583	0.767	0.044	**0.723**	79.46	4	4	F	40
584	0.626	0.032	**0.594**	60.34	4	4	F	40
585	0.517	0.018	**0.499**	46.26	4	4	F	57
586	0.734	0.028	**0.706**	76.94	4	4	M	63
587	0.681	0.052	**0.629**	65.53	4	4	F	63
588	0.517	0.1	**0.417**	34.10	3	3	M	74
589	0.491	0.055	**0.436**	36.92	3	3	F	64
590	0.417	0.021	**0.396**	30.99	3	3	M	72
591	0.35	0.061	**0.289**	15.13	3	3	F	60
592	0.413	0.022	**0.391**	30.25	3	3	F	73
593	0.931	0.025	**0.906**	106.59	4	4	F	54
594	0.48	0.049	**0.431**	36.18	3	3	F	50
595	0.761	0.082	**0.679**	72.94	4	4	F	60
596	0.709	0.091	**0.618**	63.90	4	4	M	61
597	0.461	0.06	**0.401**	31.73	3	3	F	59
598	0.819	0.062	**0.757**	84.50	4	4	F	63
599	0.897	0.041	**0.856**	99.18	4	4	M	64
600	0.525	0.028	**0.497**	45.96	3\4	3	F	41
601	0.455	0.034	**0.421**	34.70	3	3	F	47
602	0.444	0.013	**0.431**	36.18	3	3	M	40
603	0.492	0.055	**0.437**	37.07	3	3	F	62
604	0.549	0.076	**0.473**	42.40	3	3	F	63
605	0.596	0.12	**0.476**	42.85	3	3	M	71
606	0.742	0.085	**0.657**	69.68	4	4	M	73
607	0.653	0.056	**0.597**	60.78	4	4	F	70
608	0.717	0.037	**0.68**	73.09	4	4	F	52
609	0.523	0.021	**0.502**	46.70	4	4	M	65
610	0.405	0.011	**0.394**	30.69	3	3	F	58
611	0.58	0.072	**0.508**	47.59	3	3	M	54
612	0.598	0.099	**0.499**	46.26	3	3	M	70
613	0.602	0.05	**0.552**	54.11	4	4	F	54
614	0.7	0.061	**0.639**	67.01	4	4	F	70
615	0.85	0.039	**0.811**	92.51	4	4	M	64
616	0.761	0.05	**0.711**	77.68	4	4	M	55
617	0.279	0.016	**0.263**	11.27	3	3	M	67
618	0.573	0.039	**0.534**	51.45	3\4	3	F	49
619	0.564	0.074	**0.49**	44.92	3	3	F	70
620	0.704	0.132	**0.572**	57.08	3	3	M	65
621	0.485	0.092	**0.393**	30.54	3	3	M	66
622	0.409	0.014	**0.395**	30.84	3	3	M	62
623	0.76	0.053	**0.707**	77.09	4	4	F	72

624	0.473	0.053	**0.42**	34.55	3	3	F	60
625	0.502	0.06	**0.442**	37.81	3	3	F	62
626	0.199	0.01	**0.189**	0.31	1	1	F	72
627	0.804	0.07	**0.734**	81.09	4	4	F	52
628	0.94	0.106	**0.834**	95.91	4	4	F	69
629	0.854	0.069	**0.785**	88.65	4	4	F	63
630	0.826	0.072	**0.754**	84.06	4	4	F	74
631	0.467	0.048	**0.419**	34.40	2	2	F	30
632	0.934	0.053	**0.881**	102.88	4	4	F	56
633	0.913	0.041	**0.872**	101.55	4	4	M	42
634	0.821	0.05	**0.771**	86.58	4	4	M	70
**Plate 9**								
Blank	**0**	**0**	**0**		**No**	**No**		
Sub Negative	**0.073**	**0.065**	**0.008**		**No**	**No**		
Inactivated	**0.111**	**0.106**	**0.005**		**N0**	**No**		
100	**1.249**	**0.252**	**0.997**		**5**	**4**		
60	**0.822**	**0.032**	**0.79**		**4**	**4**		
30	**0.434**	**0.063**	**0.371**		**3**	**3**		
10	**0.262**	**0.04**	**0.222**		**2**	**2**		
635	0.5	0.056	**0.444**	33.91	3	3	F	53
636	0.775	0.07	**0.705**	61.72	4	4	M	57
637	0.429	0.012	**0.417**	31.03	2	2	F	71
638	0.698	0.041	**0.657**	56.61	4	4	M	56
639	0.414	0.048	**0.366**	25.59	2	2	F	53
640	0.619	0.049	**0.57**	47.34	4	3	M	57
641	0.614	0.076	**0.538**	43.93	3	3	F	20
642	0.286	0.058	**0.228**	10.89	2	2	F	61
643	0.805	0.101	**0.704**	61.62	4	4	F	18
644	0.661	0.064	**0.597**	50.21	4	4	M	65
645	0.563	0.073	**0.49**	38.81	3	3	M	70
646	0.463	0.041	**0.422**	31.56	3	3	F	31
647	1.073	0.061	**1.012**	94.44	4	4	F	51
648	0.41	0.04	**0.37**	26.02	2	2	M	71
649	0.562	0.091	**0.471**	36.78	3	3	M	1 5/12
650	0.584	0.089	**0.495**	39.34	3	3	M	2 2 /12
651	0.826	0.088	**0.738**	65.24	4	4	M	57
652	1.043	0.093	**0.95**	87.83	5	5	F	55
653	1.495	0.276	**1.219**	116.50	6	6	F	36
654	0.435	0.078	**0.357**	24.64	2	2	M	46
655	0.689	0.063	**0.626**	53.30	3	3	M	1 6/ 12
656	0.701	0.055	**0.646**	55.44	4	4	M	79
657	0.683	0.04	**0.643**	55.12	4	4	F	57
658	0.681	0.094	**0.587**	49.15	3	3	F	55

659	0.65	0.075	**0.575**	47.87	4	4	F	43
660	0.731	0.085	**0.646**	55.44	4	4	F	39
661	0.734	0.051	**0.683**	59.38	4	4	M	63
662	0.711	0.132	**0.579**	48.29	5	4	M	57
663	0.557	0.039	**0.518**	41.79	3	3	F	73
664	0.16	0.012	**0.148**	2.36	2	2	F	75
665	0.655	0.099	**0.556**	45.84	4	4	F	55
666	0.61	0.094	**0.516**	41.58	4	4	F	54
667	0.442	0.024	**0.418**	31.14	3	3	F	70
668	0.457	0.033	**0.424**	31.78	3	3	F	54
669	0.577	0.05	**0.527**	42.75	4	4	F	61
670	0.567	0.113	**0.454**	34.97	4	4	M	49
671	0.628	0.063	**0.565**	46.80	3	3	F	75
672	0.625	0.066	**0.559**	46.16	4	4	M	29
673	0.628	0.065	**0.563**	46.59	4	4	M	63
674	0.268	0.034	**0.234**	11.53	2	2	F	63
675	0.575	0.117	**0.458**	35.40	4	4	M	58
676	0.537	0.08	**0.457**	35.29	4	4	F	50
677	0.619	0.065	**0.554**	45.63	4	4	F	54
678	0.367	0.072	**0.295**	18.03	3	3	F	57
679	0.476	0.059	**0.417**	31.03	3	3	F	51
680	0.584	0.048	**0.536**	43.71	3	3	M	72
681	0.502	0.092	**0.41**	30.28	3	3	F	40
682	0.42	0.011	**0.409**	30.18	3	2	F	64
683	0.651	0.077	**0.574**	47.76	4	4	F	53
684	0.724	0.105	**0.619**	52.56	4	4	M	66
685	0.517	0.101	**0.416**	30.92	3	3	F	60
686	0.36	0.081	**0.279**	16.32	2	2	F	55
687	0.581	0.079	**0.502**	40.09	3	3	F	41
688	0.489	0.054	**0.435**	32.95	3	3	F	70
689	0.448	0.031	**0.417**	31.03	3	3	M	65
690	0.873	0.145	**0.728**	64.17	5	5	F	66
691	0.685	0.101	**0.584**	48.83	5	5	F	55
692	0.69	0.196	**0.494**	39.24	5	5	F	48
693	1.315	0.143	**1.172**	111.49	6	6	M	6
694	0.876	0.112	**0.764**	68.01	6	6	M	54
695	0.453	0.027	**0.426**	31.99	3	3	F	32
696	0.633	0.103	**0.53**	43.07	4	4	F	62
697	0.535	0.091	**0.444**	33.91	3	3	F	38
698	0.374	0.125	**0.249**	13.13	2	2	F	65
699	0.571	0.086	**0.485**	38.28	3	3	M	80
700	0.533	0.102	**0.431**	32.52	3	3	F	63
701	0.539	0.092	**0.447**	34.23	4	4	F	27

702	0.367	0.075	**0.292**	17.71	3	3	F	59
703	0.439	0.023	**0.416**	30.92	3	3	F	36
704	0.492	0.068	**0.424**	31.78	4	4	F	13
705	0.913	0.103	**0.81**	72.91	5	5	F	37
706	0.775	0.297	**0.478**	37.53	4	4	F	28
707	0.815	0.123	**0.692**	60.34	5	5	F	27
708	0.815	0.145	**0.67**	57.99	5	5	F	45
709	0.503	0.083	**0.42**	31.35	4	4	F	53
710	0.535	0.109	**0.426**	31.99	4	4	F	67
711	0.962	0.074	**0.888**	81.23	5	5	F	62
712	0.981	0.103	**0.878**	80.16	5	5	M	58
713	0.831	0.11	**0.721**	63.43	4	4	F	54
714	0.604	0.147	**0.457**	35.29	3	3	F	54
715	0.539	0.101	**0.438**	33.27	3	3	F	70
716	0.739	0.126	**0.613**	51.92	3	3	M	56
717	1.155	0.111	**1.044**	97.85	5	5	F	48
718	0.518	0.104	**0.414**	30.71	4	4	M	64
719	0.756	0.082	**0.674**	58.42	4	4	F	65
720	1.381	0.141	**1.24**	118.74	5	5	M	40
721	0.845	0.154	**0.691**	60.23	4	4	F	43
722	0.639	0.154	**0.485**	38.28	3	3	M	61
723	0.756	0.128	**0.628**	53.52	3	3	F	56
724	0.522	0.148	**0.374**	26.45	2	2	F	54
725	0.71	0.12	**0.59**	49.47	3	3	F	46
726	0.79	0.134	**0.656**	56.50	3	3	M	57
727	0.735	0.102	**0.633**	54.05	3	3	M	50
728	0.484	0.066	**0.418**	31.14	3	3	M	61
**Plate 10**								
Blank	**0**	**0**	**0**		**No**	**No**		
Sub Negative	**0.209**	**0.19**	**0.019**		**No**	**No**		
Inactivated	**0.209**	**0.162**	**0.047**		**No**	**no**		
100	**1.09**	**0.091**	**0.999**		**5**	**5**		
60	**0.716**	**0.139**	**0.577**		**4**	**4**		
30	**0.566**	**0.087**	**0.479**		**3**	**3**		
10	**0.472**	**0.073**	**0.399**		**2**	**2**		
729	0.966	0.076	**0.89**	88.99	5	5	M	56
730	0.967	0.071	**0.896**	89.83	5	5	F	49
731	0.742	0.05	**0.692**	61.07	5	5	F	52
732	1.186	0.063	**1.123**	121.84	5	5	M	58
733	1.04	0.04	**1**	104.50	5	5	M	39
734	0.756	0.052	**0.704**	62.76	5	5	F	82
735	1.064	0.064	**1**	104.50	4	4	F	52
736	1.129	0.088	**1.041**	110.28	5	5	F	61

737	1.291	0.084	**1.207**	133.68	5	5	F	50
738	1.207	0.066	**1.141**	124.38	5	5	M	62
739	1.412	0.175	**1.237**	137.91	5	5	M	63
740	0.972	0.048	**0.924**	93.78	5	5	F	55
741	1.183	0.05	**1.133**	123.25	5	5	F	56
742	1.467	0.037	**1.43**	165.13	5	5	M	67
743	0.863	0.057	**0.806**	77.14	4	4	F	67
744	1.621	0.359	**1.262**	141.44	5	5	F	59
745	1.048	0.056	**0.992**	103.37	5	5	M	65
746	0.888	0.043	**0.845**	82.64	5	5	M	44
747	1.217	0.206	**1.011**	106.05	5	5	F	57
748	0.661	0.046	**0.615**	50.21	4	4	F	65
749	0.818	0.031	**0.787**	74.46	4	4	F	50
750	1.465	0.214	**1.251**	139.89	5	5	F	57
751	0.744	0.018	**0.726**	65.86	4	4	F	63
752	0.663	0.074	**0.589**	46.55	4	4	F	76
753	1.695	0.171	**1.524**	178.38	6	6	M	65
754	0.496	0.012	**0.484**	31.74	4	4	F	68
755	0.986	0.042	**0.944**	96.60	5	5	F	67
756	0.901	0.046	**0.855**	84.05	5	5	F	54
757	0.957	0.039	**0.918**	92.93	5	5	F	60
758	0.978	0.03	**0.948**	97.16	5	5	F	33
759	0.539	0.058	**0.481**	31.32	4	4	F	65
760	0.613	0.091	**0.522**	37.10	4	4	M	61
761	1.011	0.057	**0.954**	98.01	5	5	F	59
762	0.908	0.084	**0.824**	79.68	4	5	M	70
763	0.729	0.102	**0.627**	51.90	4	4	F	38
764	1.626	0.12	**1.506**	175.84	6	6	M	62
765	1.561	0.102	**1.459**	169.22	6	6	F	53
766	1.314	0.051	**1.263**	141.58	6	6	F	24
767	0.68	0.058	**0.622**	51.20	4	4	F	62
768	1.581	0.243	**1.338**	152.15	6	6	F	59
769	1.572	0.3	**1.272**	142.85	6	6	M	55
770	1.134	0.095	**1.039**	110.00	5	5	F	54
771	1.055	0.053	**1.002**	104.78	5	5	F	65
772	1.62	0.212	**1.408**	162.02	6	6	F	19
773	0.69	0.047	**0.643**	54.16	5	5	F	62
774	1.843	0.459	**1.384**	158.64	6	6	F	51
775	1.431	0.011	**1.42**	163.72	7	7	F	25
776	1.207	0.101	**1.106**	119.44	6	6	F	74
777	1.241	0.076	**1.165**	127.76	6	6	M	50
778	1.627	0.135	**1.492**	173.87	7	7	F	36
779	0.673	0.07	**0.603**	48.52	5	5	F	64

780	1.159	0.067	**1.092**	117.47	5	5	M	66
781	1.65	0.358	**1.292**	145.67	6	6	F	74
782	1.839	0.368	**1.471**	170.91	6	6	M	46
783	1.522	0.139	**1.383**	158.50	6	6	M	48
784	0.563	0.081	**0.482**	31.46	4	4	F	60
785	1.246	0.092	**1.154**	126.21	6	6	F	43
786	1.367	0.124	**1.243**	138.76	6	6	F	50
787	2.1	0.746	**1.354**	154.41	6	6	F	40
**Plate 11**								
Blank	**0.022**	**0.022**	**0**		**No**	**No**		
Sub Negative	**0.076**	**0.045**	**0.031**		**No**	**No**		
Inactivated	**0.114**	**0.089**	**0.025**		**No**	**No**		
100	**1.201**	**0.057**	**1.144**		**5**	**6**		
60	**0.701**	**0.131**	**0.57**		**4**	**4**		
30	**0.436**	**0.024**	**0.412**		**3**	**3**		
10	**0.189**	**0.015**	**0.174**		**2**	**2**		
788	0.512	0.105	**0.407**	34.32	3	3	M	65
789	0.305	0.127	**0.178**	12.95	No	3	M	64
790	0.493	0.019	**0.474**	40.57	3	3	F	54
791	0.479	0.014	**0.465**	39.74	3	4	F	64
792	0.398	0.028	**0.37**	30.87	3	4	M	71
793	0.586	0.042	**0.544**	47.11	4	4	M	54
794	0.581	0.023	**0.558**	48.41	4	3	F	62
795	0.737	0.026	**0.711**	62.69	4	3	F	47
796	0.456	0.017	**0.439**	37.31	3	3	F	66
797	0.642	0.03	**0.612**	53.45	4	3	F	43
798	0.884	0.33	**0.554**	48.04	3	3	F	63
799	0.719	0.15	**0.569**	49.44	3	4	M	58
800	0.578	0.15	**0.428**	36.28	3	5	M	67
801	0.869	0.032	**0.837**	74.45	4	4	M	42
802	0.31	0.002	**0.308**	25.08	3	3	M	59
803	0.516	0.041	**0.475**	40.67	3	3	M	75
804	0.492	0.021	**0.471**	40.29	3	2	M	48
805	0.529	0.054	**0.475**	40.67	3	4	M	53
806	0.283	0.054	**0.229**	17.71	2	2	M	70
807	0.768	0.059	**0.709**	62.50	4	4	M	47
808	0.217	0.025	**0.192**	14.26	2	2	F	62
809	0.527	0.052	**0.475**	40.67	4	3	F	55
810	0.825	0.036	**0.789**	69.97	5	5	M	61
811	0.401	0.031	**0.37**	30.87	3	3	M	58
812	0.418	0.043	**0.375**	31.34	3	4	F	67
813	0.382	0.015	**0.367**	30.59	3	3	M	71
814	0.666	0.064	**0.602**	52.52	4	4	F	62

815	0.28	0.05	**0.23**	17.81	2	2	F	61
816	0.401	0.027	**0.374**	31.24	3	3	F	31
817	0.544	0.059	**0.485**	41.60	3	3	F	67
818	0.486	0.032	**0.454**	38.71	3	4	F	57
819	0.412	0.046	**0.366**	30.50	3	3	M	30
820	0.381	0.009	**0.372**	31.06	3	3	F	37
821	0.57	0.022	**0.548**	47.48	3	3	F	57
822	0.902	0.063	**0.839**	74.64	4	4	F	65
823	0.651	0.022	**0.629**	55.04	4	4	M	54
824	0.633	0.028	**0.605**	52.80	4	4	M	33
825	0.727	0.115	**0.612**	53.45	4	4	F	67
826	0.271	0.042	**0.229**	17.71	3	3	M	73
827	0.409	0.037	**0.372**	31.06	3	3	M	74
828	0.412	0.011	**0.401**	33.76	3	4	F	65
829	0.157	0.034	**0.123**	7.82	2	2	M	67
830	0.518	0.008	**0.51**	43.93	4	4	M	78
831	0.471	0.016	**0.455**	38.80	3	3	M	52
832	0.907	0.048	**0.859**	76.50	4	4	F	70
833	0.808	0.167	**0.641**	56.16	4	4	F	75
834	0.35	0.034	**0.316**	25.83	2	2	F	43
835	0.728	0.04	**0.688**	60.54	5	4	M	81
836	0.531	0.109	**0.422**	35.72	4	3	F	54
837	0.595	0.042	**0.553**	47.95	4	3	F	10
838	1.143	0.505	**0.638**	55.88	4	3	F	58
839	0.38	0.01	**0.37**	30.87	4	3	M	61
840	0.494	0.048	**0.446**	37.96	3	3	F	55
841	0.602	0.084	**0.518**	44.68	4	4	F	65
842	0.683	0.187	**0.496**	42.63	3	3	F	52
843	0.486	0.066	**0.42**	35.54	3	3	F	55
844	0.57	0.038	**0.532**	45.99	3	2	F	14
845	0.451	0.058	**0.393**	33.02	3	4	F	53
846	0.591	0.023	**0.568**	49.35	4	3	F	20
847	0.504	0.034	**0.47**	40.20	3	3	F	38
848	0.546	0.101	**0.445**	37.87	3	3	M	64
849	0.692	0.078	**0.614**	53.64	3	3	F	46
850	0.53	0.052	**0.478**	40.95	3	3	M	54
851	0.543	0.112	**0.431**	36.56	3	3	F	50
852	0.595	0.045	**0.55**	47.67	3	4	F	42
853	0.518	0.044	**0.474**	40.57	3	3	F	41
854	0.522	0.023	**0.499**	42.91	3	3	F	58
855	0.526	0.051	**0.475**	40.67	3	3	M	90
856	0.561	0.069	**0.492**	42.25	3	3	M	75
857	0.484	0.098	**0.386**	32.36	3	3	M	64

858	0.44	0.059	**0.381**	31.90	3	3	F	49
859	0.508	0.086	**0.422**	35.72	3	3	F	62
860	0.929	0.144	**0.785**	69.60	4	4	F	19
861	0.485	0.051	**0.434**	36.84	3	3	M	53
862	0.477	0.043	**0.434**	36.84	3	3	F	53
863	1.04	0.089	**0.951**	85.09	5	5	F	3y9m
864	0.876	0.11	**0.766**	67.82	4	4	F	3y7m
865	0.579	0.175	**0.404**	34.04	3	3	F	72
866	0.793	0.068	**0.725**	64.00	4	4	F	14
867	0.931	0.104	**0.827**	73.52	4	4	M	50
868	1.376	0.181	**1.195**	107.86	5	5	F	72
869	1.333	0.102	**1.231**	111.22	5	5	F	65
870	0.693	0.042	**0.651**	57.09	4	4	M	13
871	1.324	0.047	**1.277**	115.51	5	5	F	14
**Plate 12**								
Blank	**0**	**0**	**0**		**No**	**No**		
Sub Negative	**0.085**	**0.067**	**0.018**		**No**	**No**		
Inactivated	**0.036**	**0.005**	**0.031**		**No**	**No**		
100	**1.4**	**0.29**	**1.11**		**6**	**7**		
60	**0.569**	**0.082**	**0.487**		**3**	**3**		
30	**0.363**	**0.04**	**0.323**		**2**	**2**		
10	**0.337**	**0.208**	**0.129**		**1**	**1**		
872	1.191	0.023	**1.168**	109.13	6	6	F	40
873	1.182	0.019	**1.163**	108.68	6	6	F	57
874	0.83	0.037	**0.793**	75.32	5	5	F	67
875	0.934	0.021	**0.913**	86.14	6	6	M	52
876	0.853	0.295	**0.558**	54.13	3	3	M	64
877	1.118	0.029	**1.089**	102.01	5	5	F	65
878	1.468	0.05	**1.418**	131.67	7	7	F	44
879	0.795	0.13	**0.665**	63.77	4	4	M	84
880	0.6	0.028	**0.572**	55.39	5	5	F	36
881	0.997	0.103	**0.894**	84.42	5	5	F	64
882	1.239	0.202	**1.037**	97.32	6	6	M	49
883	1.239	0.125	**1.114**	104.26	7	7	F	65
884	0.533	0.042	**0.491**	48.08	3	3	M	53
885	1.116	0.115	**1.001**	94.07	5	5	F	37
886	1.207	0.061	**1.146**	107.15	6	6	F	65
887	1.171	0.288	**0.883**	83.43	5	5	F	64
888	1.315	0.015	**1.3**	121.03	6	6	F	57
889	1.065	0.011	**1.054**	98.85	5	5	F	26
890	0.929	0.015	**0.914**	86.23	4	4	M	36
891	0.525	0.011	**0.514**	50.16	3	3	F	48
892	0.779	0.047	**0.732**	69.82	3	4	F	64

893	1.123	0.253	0.87	82.26	6	6	F	64
894	1.547	0.115	1.432	132.93	7	7	F	52
895	0.698	0.032	0.666	63.86	5	5	F	68
896	0.406	0.048	0.358	36.09	6	6	M	49
897	0.987	0.039	0.948	89.29	5	5	F	58
898	0.363	0.079	0.284	29.42	2	2	M	47
899	1.257	0.154	1.103	103.27	7	7	F	65
900	0.631	0.074	0.557	54.04	3	3	F	57
901	1.463	0.097	1.366	126.98	7	7	F	65
902	1.585	0.22	1.365	126.89	7	7	M	64
903	1.278	0.187	1.091	102.19	6	6	F	73
904	0.873	0.013	0.86	81.36	6	6	M	55
905	0.833	0.013	0.82	77.75	5	5	M	82
906	0.856	0.01	0.846	80.09	4	4	M	63
907	1.516	0.055	1.461	135.55	7	7	F	27
908	0.585	0.075	0.51	49.80	3	3	M	78
909	0.429	0.051	0.378	37.89	2	2	F	70
910	0.427	0.035	0.392	39.16	3	3	M	66
911	0.621	0.187	0.434	42.94	3	3	F	65
912	0.712	0.024	0.688	65.85	4	4	M	61
913	0.677	0.022	0.655	62.87	4	4	F	62
914	0.49	0.035	0.455	44.84	3	3	F	63
915	0.32	0.042	0.278	28.88	2	2	F	55
916	0.326	0.039	0.287	29.69	2	2	F	67
917	0.269	0.061	0.208	22.57	3	3	F	62
918	1.584	0.068	1.516	140.51	7	7	M	53
919	0.554	0.063	0.491	48.08	4	4	M	55
920	0.589	0.025	0.564	54.67	4	4	M	66
921	1.482	0.145	1.337	124.37	7	7	F	55
922	0.632	0.025	0.607	58.54	5	5	M	56
923	1.599	0.084	1.515	140.42	7	7	M	68
924	1.008	0.213	0.795	75.50	4	4	F	62
925	0.727	0.093	0.634	60.98	4	4	F	50
926	0.345	0.066	0.279	28.97	3	3	F	52
927	0.445	0.106	0.339	34.38	3	3	M	70
928	0.536	0.063	0.473	46.46	4	4	M	69
**Plate 13**								
Blank	0.01	0	0.01		No	No		
Sub Negative	0.181	0.061	0.12		No	No		
Inactivated	0.235	0.222	0.013		No	No		
100	1.308	0.338	0.97		5	5		
60	0.742	0.083	0.659		3	3		
30	0.304	0.025	0.279		2	2		

10	**0.275**	**0.019**	**0.256**		**2**	**2**		
929	1.232	0.023	**1.209**	125.42	5	5	F	66
930	1.036	0.025	**1.011**	103.06	5	5	F	15
931	1.032	0.041	**0.991**	100.80	5	5	M	53
932	1.646	0.052	**1.594**	168.88	6	6	M	21
933	1.317	0.066	**1.251**	130.16	5	5	F	66
934	0.891	0.072	**0.819**	81.39	4	4	F	70
935	1.071	0.057	**1.014**	103.40	5	5	F	59
936	0.912	0.041	**0.871**	87.26	5	5	M	55
937	1.476	0.034	**1.442**	151.72	5	5	F	42
938	1.111	0.036	**1.075**	110.29	5	5	F	60
939	1.599	0.132	**1.467**	154.54	5	5	M	45
940	0.494	0.058	**0.436**	38.15	3	3	F	74
941	0.894	0.035	**0.859**	85.90	5	5	F	42
942	0.771	0.098	**0.673**	64.90	4	4	F	71
943	0.767	0.04	**0.727**	71.00	4	4	F	45
944	1.241	0.01	**1.231**	127.90	5	5	F	37
945	0.654	0.042	**0.612**	58.02	4	5	F	33
946	0.946	0.023	**0.923**	93.13	5	4	F	57
947	0.922	0.038	**0.884**	88.72	5	4	M	36
948	1.386	0.065	**1.321**	138.06	5	4	F	56
949	0.396	0.014	**0.382**	32.05	3	3	F	67
950	0.696	0.07	**0.626**	59.60	4	4	F	42
951	1.037	0.045	**0.992**	100.92	5	5	M	75
952	0.874	0.055	**0.819**	81.39	4	4	M	65
953	1.031	0.193	**0.838**	83.53	5	5	F	52
954	1.204	0.018	**1.186**	122.82	5	5	M	68
955	0.738	0.052	**0.686**	66.37	4	4	F	55
956	0.853	0.032	**0.821**	81.61	5	5	F	58
957	1.155	0.01	**1.145**	118.19	5	5	F	61
958	0.905	0.086	**0.819**	81.39	5	5	F	49
959	1.156	0.047	**1.109**	114.13	5	5	F	73
960	0.906	0.164	**0.742**	72.69	5	5	F	80
961	0.987	0.036	**0.951**	96.29	5	4	F	54
962	1.004	0.138	**0.866**	86.69	5	5	M	68
963	0.467	0.011	**0.456**	40.40	3	3	F	34
964	0.931	0.07	**0.861**	86.13	5	5	F	50
965	1.03	0.027	**1.003**	102.16	5	5	F	62
966	0.738	0.081	**0.657**	63.10	4	5	F	25
967	0.677	0.046	**0.631**	60.16	4	4	M	72
968	0.923	0.064	**0.859**	85.90	5	4	M	55
969	0.535	0.023	**0.512**	46.73	4	5	F	70
970	0.612	0.02	**0.592**	55.76	4	4	M	70

971	0.899	0.039	**0.86**	86.01	5	4	M	59
972	0.771	0.016	**0.755**	74.16	4	4	M	55
973	0.744	0.055	**0.689**	66.71	4	4	F	50
974	0.8	0.048	**0.752**	73.82	4	4	F	64
975	0.979	0.055	**0.924**	93.24	5	5	F	56
976	0.849	0.039	**0.81**	80.37	4	4	F	63
977	0.842	0.029	**0.813**	80.71	4	4	M	55
978	1.419	0.103	**1.316**	137.50	5	4	M	58
979	0.709	0.038	**0.671**	64.68	4	4	F	42
980	0.581	0.026	**0.555**	51.58	4	4	F	67
981	0.443	0.053	**0.39**	32.95	3	3	F	79
982	0.997	0.148	**0.849**	84.77	5	5	M	60
983	0.967	0.046	**0.921**	92.90	5	4	M	54
984	0.696	0.053	**0.643**	61.52	4	4	F	52
985	1.128	0.035	**1.093**	112.32	5	5	F	32
986	1.14	0.043	**1.097**	112.77	5	5	M	65
987	1.145	0.088	**1.057**	108.26	5	4	F	45
988	1.466	0.045	**1.421**	149.35	5	4	F	58
989	0.689	0.043	**0.646**	61.85	4	4	F	36
990	1.27	0.117	**1.153**	119.09	5	5	F	55
991	0.396	0.015	**0.381**	31.94	3	2	F	45
992	0.693	0.077	**0.616**	58.47	4	4	F	74
993	0.405	0.033	**0.372**	30.92	3	3	F	67
994	0.723	0.036	**0.687**	66.48	4	4	F	50
995	0.82	0.019	**0.801**	79.35	4	4	F	54
996	0.654	0.034	**0.62**	58.92	4	3	M	65
997	1.085	0.084	**1.001**	101.93	5	5	M	59
998	0.926	0.121	**0.805**	79.81	5	5	F	49
999	0.437	0.05	**0.387**	32.61	3	2	F	60
1000	0.768	0.08	**0.688**	66.60	4	5	F	57
1001	1.016	0.055	**0.961**	97.42	5	4	F	33
1002	1.33	0.214	**1.116**	114.92	5	5	F	57
1003	0.996	0.076	**0.92**	92.79	5	4	M	66
1004	0.433	0.058	**0.375**	31.26	3	3	F	65
1005	0.628	0.089	**0.539**	49.77	4	4	M	56
1006	0.855	0.085	**0.77**	75.85	4	5	M	42
1007	0.835	0.085	**0.75**	73.60	4	3	M	61
1008	0.954	0.087	**0.867**	86.80	5	4	M	45
1009	0.564	0.033	**0.531**	48.87	4	4	M	71
1010	0.659	0.076	**0.583**	54.74	4	5	M	62
1011	0.727	0.083	**0.644**	61.63	4	4	F	72
1012	0.98	0.131	**0.849**	84.77	5	4	M	55
**Plate 14**								

Blank	**0**	**0**	**0**		**No**	**No**		
Sub Negative	**0.163**	**0.108**	**0.055**		**No**	**No**		
Inactivated	**0.103**	**0.041**	**0.062**		**No**	**No**		
100	**1.236**	**0.313**	**0.923**		**5**	**5**		
60	**0.7**	**0.061**	**0.639**		**4**	**4**		
30	**0.556**	**0.081**	**0.475**		**4**	**4**		
10	**0.227**	**0.054**	**0.173**		**No**	**No**		
1013	0.947	0.105	**0.842**	85.47	5	5	F	72
1014	0.99	0.023	**0.967**	100.79	5	4	F	45
1015	0.593	0.024	**0.569**	52.02	3	3	F	68
1016	0.714	0.028	**0.686**	66.36	4	4	M	67
1017	1.131	0.054	**1.077**	114.27	5	5	F	67
1018	0.643	0.038	**0.605**	56.43	4	4	F	54
1019	0.835	0.029	**0.806**	81.06	4	4	F	63
1020	0.913	0.02	**0.893**	91.72	5	5	F	80
1021	1.103	0.017	**1.086**	115.37	5	5	F	63
1022	0.884	0.023	**0.861**	87.80	4	4	F	56
1023	0.691	0.015	**0.676**	65.13	4	4	F	50
1024	0.901	0.014	**0.887**	90.99	5	5	F	65
1025	0.994	0.027	**0.967**	100.79	5	5	M	43
1026	0.871	0.029	**0.842**	85.47	4	4	F	70
1027	0.343	0.045	**0.298**	18.82	3	3	M	31
1028	1.123	0.01	**1.113**	118.68	5	5	M	78
1029	1.398	0.033	**1.365**	149.56	5	5	M	43
1030	0.9	0.035	**0.865**	88.29	5	4	M	59
1031	0.845	0.015	**0.83**	84.00	4	4	M	40
1032	0.763	0.021	**0.742**	73.22	4	4	F	47
1033	0.596	0.018	**0.578**	53.12	4	4	F	38
1034	0.709	0.058	**0.651**	62.07	4	4	F	40
1035	0.615	0.014	**0.601**	55.94	4	4	M	61
1036	0.885	0.035	**0.85**	86.45	4	4	F	57
1037	1.019	0.016	**1.003**	105.20	5	5	F	30
1038	1.185	0.025	**1.16**	124.44	5	4	M	52
1039	0.649	0.017	**0.632**	59.74	4	4	F	58
1040	1.317	0.03	**1.287**	140.00	5	5	F	59
1041	0.256	0.019	**0.237**	11.34	2	2	M	68
